# A multifunctional organelle coordinates phagocytosis and chlorophagy in a marine eukaryote phytoplankton *Scyphosphaera apsteinii*

**DOI:** 10.1111/nph.20388

**Published:** 2025-03-04

**Authors:** Julie A. Koester, Oren Fox, Elizabeth Smith, Madison B. Cox, Alison R. Taylor

**Affiliations:** ^1^ Department of Biology and Marine Biology University of North Carolina Wilmington 601 South College Road Wilmington NC 28403 USA

**Keywords:** autophagy, chlorophagy, coccolithophore, lysosome, mixotrophy, organelle, phagocytosis, vacuole

## Abstract

- Mixotrophy via phagocytosis can have profound consequences for the survival of marine phytoplankton and the efficiency of carbon transfer in marine systems. Little is known about the cellular mechanisms that underly nutrient acquisition via prey uptake and processing in mixotrophic phytoplankton.
- We used confocal microscopy, flow cytometry, and electron microscopy to assess phagocytosis and intracellular prey processing in the diploid calcifying coccolithophore *Scyphosphaera apsteinii*. Bioinformatic analysis was performed to develop a working model of the pathways that likely converge to regulate mixotrophic nutrition and autophagy.
- We found cells ingested proxy (up to 2 μm diameter) and natural (bacteria and cyanobacteria) prey particles that are processed within a single, prominent acidic vacuole detected in 80–100% of cells during exponential growth. This organelle was constitutive in cells through all growth phases to late stationary and is inherited when cells divide. Chloroplast fragments localized to this digestive organelle. A distinct, nonacidic vacuole containing polyphosphate was also identified in cells with ingested particles.
- We conclude a novel acidic organelle plays a multifunctional catabolic role in both mixotrophic nutrition (phagotrophy) and autophagy (chlorophagy). This discovery illustrates the dynamic nutritional strategies that marine phytoplankton, such as coccolithophores, have evolved to acquire and conserve nutrients.

## Introduction

Haptophyte microalgae, including biomineralizing coccolithophores and naked flagellates, comprise 30–50% of the standing stock of microbial primary producers in the world's ocean (Liu *et al*., 2009). As mixotrophs, haptophyte flagellates are responsible for, on average, 40% of bacterivory in oligotrophic ecosystems (Unrein *et al*., 2014). Mixotrophy is a collection of functional physiological traits defined by a combination of autotrophic and phagotrophic carbon acquisition (Raven *et al*., 2009; Flynn *et al*., 2019) distinct from osmotrophy, which is the uptake of dissolved organic molecules (Flynn *et al*., 2013). Mixoplankton are important drivers of carbon flow in pelagic microbial communities (Mitra *et al*., 2014, 2016; Ward & Follows, 2016), with significant contributions to bacterivory and carbon transfer by pico‐ and nanoplankton (Zubkov & Tarran, 2008). Mixotrophy also allows for life‐history, phenotypic, and habitat flexibility, including the ability to thrive in oligotrophic regions or survive subphotic conditions and periods of darkness (Brutemark & Granéli, 2011; Anderson *et al*., 2018; Wilken *et al*., 2020). Experimental and modeling studies suggest mixotrophy traits, including increased phagotrophy, may be favored under ocean warming scenarios, for example if metabolic rate responses of phagotrophy are greater than those of photosynthesis (Gonzalez *et al*., 2022; Lepori‐Bui *et al*., 2022).

Studies directly testing phagocytosis in haptophytes have been conducted primarily on motile representatives (Anderson *et al*., 2018), including 3 species from the calcifying subclass Calcihaptophycidae (de Vargas *et al*., 2007), the coccolithophores (Parke & Adams, 1960; Houdan *et al*., 2006; Avrahami & Frada, 2020). In addition to two flagella, motile haptophytes have a characteristic haptonema, a unique microtubule‐based appendage located between the flagella. The haptonema intercepts small prey particles in the flagella‐driven feeding currents and deposits them on the posterior portion of the cell, opposite the flagellar and haptonemal roots, where phagocytosis occurs (Parke & Adams, 1960; Kawachi *et al*., 1991; Kawachi & Inouye, 1995; Dölger *et al*., 2017). By contrast, the toxic species *Prymnesium patellifera* appears to immobilize or kill large prey before engulfing them by pseudopodia that also form at the posterior pole (Tillmann, 1998). Additionally, putatively flagellated and mixotrophic heterococcolith‐bearing species have been described from the fossil record (Gibbs *et al*., 2020) based on the arrangement of interlocking coccoliths around a regularly shaped opening that would surround the flagella and haptonema. Extant members of the Syracophaerales and some Zygodiscales (e.g. *Helicosphaera* spp.) have similar hetercoccolith arrangments, are flagellated in both the haploid and diploid phases, and are ecologically active with distributions along seasonal and environmental gradients (Šupraha *et al*., 2016; D'Amario *et al*., 2017).

The haplodiplontic life history of coccolithophores links motile, holococcolith‐bearing haploids and nonmotile heterococcolith‐bearing diploids (Houdan *et al*., 2004; Young *et al*., 2005), suggesting that mixotrophy is likely widespread across the haptophytes. However, in the absence of flagella, a haptonema, and nonoverlapping holococcoliths to enable phagotrophy, nonmotile diploids encased in interlocking heteroccoliths were thought to confine their heterotrophic nutrition to osmotrophy through the uptake of species‐specific suites of dissolved organic sources of nitrogen and phosphorus for growth (Benner & Passow, 2010; Godrijan *et al*., 2020). Transcriptomic studies of both haploid and diploid *Gephyrocapsa huxleyi* (as *Emiliania huxleyi*) indicated that they possess the molecular machinery necessary to phagocytose and enzymatically degrade potential prey particles (Rokitta *et al*., 2011); this was corroborated by low levels of phagotrophy (Ye *et al*., 2024), despite recent genetic modeling suggesting that *G. huxleyi* is purely autotrophic (Koppelle *et al*., 2022). Limited phagotrophy has been reported in 3 additional nonmotile species clad in interlocking heterococcoliths (Avrahami & Frada, 2020).

*Scyphosphaera apsteinii* Lohmann lies within the order Zygodiscales (Young & Bown, 1997). The coccosphere of nonmotile, heterococcolith‐bearing *S. apsteinii* is formed by two types of noninterlocking coccoliths, plate‐like muroliths and urn‐shaped lopadoliths, underlain by a layer of overlapping body scales. The chloroplasts are large with lobes and finger‐like extensions. The phylogenetic placement, structure, and amenability to culture has made *S. apsteinii* a model organism in studies of calcification and membrane physiology (Drescher *et al*., 2012; Durak *et al*., 2016; Langer *et al*., 2023). During those studies, decalcified *S. apsteinii* were observed using lamellipodia to move across a Petri dish and to interact with particles in the media, suggesting that it had the capacity for phagocytosis (see Supporting Information Video S1). Here, we comprehensively tested calcified *S. apsteinii* for phagocytosis and the physiological status of the hypothesized digestive vacuole using functional assays, relevant prey and visualization of ingestion, particle processing, and ultrastructure of the organelle. During these experiments, we unexpectedly observed chlorophagy. Both prey and chloroplast fragments were processed by the same prominent acidic vacuole that was constitutively expressed and was conserved throughout the cell cycle. We conclude that the vacuole is a novel multifunctional organelle likely playing an essential role in nutrient acquisition and recycling.

## Materials and Methods

### Approach

Phagocytosis was tested in *Scyphosphaera apsteinii* (Lohmann) by exposing cultured cells to fluorescently labeled beads, engineered bacterial pH sensors, and marine cyanobacteria and fluorescently labeled heterotrophic bacteria. Phagocytosis assays were assessed using confocal microscopy. The physiological state and ultrastructure of the multifunctional vacuole were determined using confocal microscopy, flow cytometry, and transmission electron microscopy. A conceptual model was constructed using the resulting data and publicly available gene sequences for *S. apsteinii* and the model coccolithophore, *G. huxleyi* (Lohmann) Reinhardt.

### Cultures and maintenance

Diploid *S. apsteinii* (strain RCC1456) and *Synechococcus* Nägeli sp. (strain CCMP1768) were grown in 40 ml batch cultures of sterilized Gulf Stream seawater supplemented with LH nutrients, trace metals, and vitamins; LH is a modified L1 medium (Fowler *et al*., 2015). Cultures were maintained at 100 μmol photons m^−2^ s^−1^ on a 14 : 10, light : dark cycle at 16°C. Growth curves for *S. apsteinii* were established by cell counting a minimum of 300 cells per replicate using a Sedgewick–Rafter chamber. The rate of increase was determined (*r* = ln(*P*_t1_/*P*_t0_)/(*t*_1_–*t*_0_)), where *P* = cell density at times *t*_0_ and *t*_1_, from which the doublings per day (*k* = *r*/0.6931), and the doubling time (*t*_d_ = 0.6931/*r*) were calculated. Maximum growth rates (*r*) of batch cultures in LH media ranged from 0.29–0.43. Frequency of subculturing for *S. apsteinii* was typically every 10 d to ensure maintenance of exponential‐phase cultures. Subcultures were made by pipetting a 2–5 ml aliquot of cells into 40–250 ml fresh media. For *Synechococcus* sp., 40 μl of cells were transferred into 40 ml fresh media every 2–3 wk.

### Enrichment of co‐occurring bacteria

Heterotrophic bacteria from the nonaxenic *S. apsteinii* cultures were enriched to produce a stock of fluorescently labeled prey for feeding experiments. A 30 ml volume of late‐exponential *S. apsteinii* culture was gently resuspended by vortexing and then pelleted at 80 RCF in a sterile 50 ml conical centrifuge tube. After centrifugation, 5 ml of the supernatant was pipetted to another 50 ml tube containing 30 ml precipitate‐free yeast extract marine broth (BD Biosciences Heterotrophic Marine Broth 2216) and incubated in the dark at room temperature for 10 d. The resulting heterotrophic bacteria were harvested through centrifugation at 3234 RCF with the final pellet of cells resuspended in 250 ml LH media, sealed with Parafilm, and stored in the dark at 4°C until use. Bacterial density was determined using a Molecular Devices SpectraMax M2^e^ spectrofluorometer to measure optical density at *λ* = 600 nm (OD_600_).

### Preparation of labeled prey particles

Carboxylate‐modified, thus negatively charged, Nile Red (NR) or Yellow‐Green (Y) FluoSphere beads (Invitrogen: 1.0 μm NR #F8819, 0.5 μm Y #F8813, 1.0 μm Y #F8823, 2.0 μm Y #F8827) were washed to remove sodium azide preservative by aliquoting 1.0 ml of the beads into a 1.5 ml microcentrifuge tube and sonicated for 5 min. Particles were then centrifuged (5 min, 13 000 RCF) and resuspended in 1.0 ml LH media 3 times before sonicating again for 5 min. Methylcellulose‐treated microspheres were prepared by centrifugation of beads as above and replacing the supernatant with nanopure water at least 3 times before replacing with 0.2% methylcellulose. Beads in methylcellulose were sonicated and vortexed to mix thoroughly and uniformly coat the beads before being rinsed by further centrifugation steps and resuspended in LH media.

Acidotropic pHrodo Red *Escherichia coli* bioparticles (pHrodo *E. coli*: Invitrogen #P35361, *c*. 1–1.5 μm in diameter) were rehydrated with LH media or unamended Gulf Stream seawater buffered with 1 mM HEPES (pH 8.0) allowing manipulation of the extracellular pH and validation of the pH‐sensitive fluorescence for batches of pHrodo *E. coli* before feeding experiments. This insured fluorescence of pHrodo *E. coli* was induced only in the acidic digestive compartment of *S. aptsteinii*. pHrodo *E. coli* were sonicated and vortexed until adequate separation of the cell pellet was verified by light microscopy. Before use, pH responsiveness was validated on each batch of pHrodo with confocal imaging at LH media pH 4.0, 6.5, and 8.0. Particles were stored in the dark at 4°C for no longer than 11 d as recommended by manufacturer.

Ingestion of the marine cyanobacterium *Synechococcus sp*. and enriched co‐occurring heterotrophic bacteria were tracked by staining the bacteria with a 0.001% solution of the cell‐permeant nucleic acid dye Acridine Orange (AO). The staining solution was added directly to the suspended *Synechococcus* cultures, whereas 10 ml aliquots of heterotrophic bacteria were sonicated for 10 min to break up large aggregates before being pelleted at 3200 RCF and resuspended in the AO solution for 20 min. Stained cells were then washed by pelleting and resuspending in LH media until there was no residual AO stain left in solution (*c*. 15–20 times) before adding to experimental replicates of *S. apsteinii*.

### Phagocytosis assays

Unless otherwise noted, *S. apsteinii* cells were sampled at mid to late‐exponential phase between 5.0 × 10^3^ and 1.0 × 10^4^ cells ml^−1^. Assays were done using 3 independent culture replicates from which 5 ml aliquots were transferred to a 7 ml polystyrene sample vial (Sterilin Bijou, Thermo Fisher) and incubated with prey particles for the indicated time. FluoSphere beads and pHrodo *E. coli* were added at a final ratio of *c*. 5000 particles per *S. apsteinii* cell (*c*. 5 × 10^7^ feeding particles ml^−1^) and incubations lasted 1–24 h. Replicates were scored for phagotrophy during the day, commencing at mid‐light cycle. The 24 h time course experiment was repeated 3 times. Incubations to investigate the effect of bead size and surface properties were for 24 h. Acridine Orange‐stained *Synechococcus* sp. or heterotrophic bacteria were added at final densities of *c*. 2 × 10^7^ bacteria cells ml^−1^, a ratio of *c*. 5 × 10^3^ bacteria per *S. apsteinii* cell, and incubated for 12 h. Experimental samples were briefly mixed by inverting the vial at least twice during the feeding trials in order to maximize interaction of prey particles and nonmotile *S. apsteinii* cells. Ecological concentrations of picoplankton (< 2 μm) vary greatly over spatial and temporal scales and while the concentrations used for these assays have been observed in pelagic systems, they are 1–2 orders of magnitude greater than typically enumerated in bulk seawater samples (Cavender‐Bares *et al*., 2001).

### Brightfield and confocal imaging of acidic vacuole and prey uptake

Lysotracker Green (LTG, Invitrogen #L7526), and in some cases Lysotracker Red (LTR, Invitrogen #L7528) dye was used to determine the proportion of cells with a prominent acidic vacuole. The LTG was tested on cells using a combination of flow cytometry and confocal microscopy, resulting in the selection of 50 nM Lysotracker in LH media as the working concentration for all subsequent experiments (Fig. S1). *S. apstenii* cells were incubated in the presence of 50 nM LTG for 30 min, after which cells were partially decalcified for 30–60 min by replacing the LTG‐LH media with Ca^2+^‐free artificial sea water supplemented with 20 mM EGTA to visualize the labeled vacuoles clearly (Taylor *et al*., 2007). The decalcification solution was replaced with fresh culture media to stop decalcification and maintain cell health for imaging.

Confocal microscopy was performed either using an inverted Olympus FV 1000 (Evident Scientific, Waltham, MA, USA) or inverted Leica SP8 confocal laser scanning microscope (Leica Microsystems, Deerfield, IL, USA) with Fluoview and Leica LASX acquisition and analysis software, respectively. *Scyphosphaera apsteinii* chlorophyll (Chl) was imaged using excitation/emission wavelengths 488/660–730 nm, Nile Red FluoSpheres at 488/585–605 nm, Yellow‐Green FluoSpheres at 458 nm/500–530 nm, LTG at 488/500–545 nm, LTR at 552/570–630 nm, pHrodo *E. coli* at 543/590–630 nm and Acridine Orange at 488/540–560 nm. Differential interference contrast (DIC) images were simultaneously acquired with a transmitted light detector. Scan settings were typically; 2–5% laser power using a scan format of 1024 by 1024 pixels and dwell time of 600 ns, a line averaging of 4, and a pinhole aperture of 1 or 1.5 Airy disc units. High‐numerical aperture oil immersion objective lenses, ×40 and ×60, were used, resulting in an optical thickness of 1.04 or 0.90 μm, respectively. Photomultiplier detector voltages were typically 530 V for Chl and between 565 and 675 V for the channels collecting fluorescent probe signals. Pixel saturation was set to maximize contrast between autofluorescence of the acidic vacuole (attributed to degradation of photosynthetic pigments) and target fluorophores (Fig. S1a–d). Labeled vacuoles were visualized by acquiring a 5‐frame z‐stack of each field of view (FOV) from the bottom of the coverslip to roughly the top of the cells. The diameter of the acidic vacuole was measured at the widest point observed in the Z‐stack of images of individual cells from replicate cultures. Additional brightfield and DIC images of cells were acquired using a Leica Thunder Imager (Leica Microsystems, Deerfield, IL, USA).

### Polyphosphate imaging

Exponentially growing *S. apsteinii* cultures (control and those treated with 1 μm Nile Red FluoSphere beads) were sampled in 2 ml aliquots and centrifuged at < 500 RCF for 5 min. Cells were suspended and fixed in a final concentration of 1% glutaraldehyde in 12.5 mM HEPES buffered LH media for 5 min. After centrifugation and removal of the supernatant, cells were further permeabilized in 1 ml 0.3% Triton‐X for 10 min after which cells were pelleted and washed twice with LH media. Cells were gently resuspended with a pipette and plated onto coverslip dishes and decalcified in 20 mM EGTA in HEPES buffered Ca^2+^‐free artificial seawater for 20–30 min after which the EGTA solution was replaced with 20 μg ml^−1^ DAPI in PBS and cells were incubated for 10 min. The DAPI solution was removed and replaced with fresh PBS for imaging. A two‐sequence imaging protocol was used to first image DAPI with the 405 nm laser and emission detection of 420–450 nm and 500–550 nm for DNA and polyphosphate (polyP), respectively. The second sequence excited the Chl (488/650–750 nm) and allowed for acquisition of DIC images.

### Flow‐cytometric analyses

Flow cytometry was conducted using a BD FACSCelesta™ Flow Cytometer (BD‐Biosciences, Malpitas, CA, USA) and data was acquired with FACSDiva software. A 488 nm excitation laser and 530/30 nm and 695/40 nm emission filters were used for LTG and Chl fluorescence signals, respectively (Fig. S1e–h). 10 000 events were collected per sample. Differences in red Chl fluorescence, side scatter, and forward scatter were used to differentiate *S. apsteinii* cells from bacteria and cellular debris, and green LTG fluorescence was used to differentiate between stained and unstained cells. Data was visualized using freely available Flowing Software 2.5.1 (Perttu Terho at Turku BioSciences, Finland) or FCSalyzer (Dr Sven Mostböck). Populations were gated using an unstained sample to set the control gate and then determining the gate for stained cells that had a significantly higher average green fluorescence. Flow cytograms for control and stained cell populations were transformed into histograms and overlaid for comparison (Fig. S1g).

### Transmission electron microscopy

Mid‐exponential phase *S. apsteinii* cultures (40 ml) were incubated in triplicate with or without 1 μm Nile Red FluoSphere beads for 24 h. Bead ingestion was verified before TEM processing by imaging live cells in z‐stacks of 2 μm increments with confocal and transmitted light, differential interphase (DIC) as described above (Fig. S2a). For TEM, control and FluoSphere‐treated cells were fixed in LH media with 2.5% glutaraldehyde at 4°C for 1 h before centrifugation at 1000 ***g*** for 10 min. Pellets were washed with 0.2 M sodium cacodylate buffer pH 7.4 three times before and after a 1 h secondary fixation with 1% osmium tetroxide in 0.2 M cacodylate buffer. Pellets were dehydrated through an ethanol series (50%, 75%, 95%, and 2 × 100%) before embedding in Spurr's resin and cured overnight at 70°C. Resin‐embedded cells were sectioned using a diamond knife (Micro Engineering Inc., Huntsville, TX, USA) and Reichert‐Jung Ultra‐cut E Microtome (Labequip Ltd, Markham, ON, Canada). Sections were mounted on Formvar‐coated 200‐mesh copper grids and stained for 15 min each in uranyl acetate (2% w/v in 50% v/v ethanol) and Reynold's lead citrate with washes of nanopure water after each stain. Sections were imaged using a TEM equipped with an Eagle Digital Camera (FEI Tecnai Spirit BioTwin; FEI, Hillsboro, OR, USA) using beam acceleration of 80 keV.

### Bioinformatic analysis

The genetic basis of conserved phagotrophic and autotrophic pathways in coccolithophores, especially *S. apsteinii*, was explored using publicly available resources. The *S. apsteinii* transcriptome from strain RCC1455 was generated during the Marine Microbial Eukaryotic Transcriptome Sequencing Project (Keeling *et al*., 2014) as MMETSP1333 and NCBI BioSample: SAMN02740197 and we used the v.2 re‐assembly (Johnson *et al*., 2018). The nucleotide sequences were translated using TransDecoder (v.5.3.0). Translated transcripts (peptides) from *S. apsteinii* were annotated for putative function using blastp to search for similar sequences against UniProt Swiss‐Prot peptides (May 2018 release) and for KEGG Orthology (KO) numbers using GhostKoala (v.2.2; Kanehisa *et al*., 2016) because the MMETSP transcriptomes were classified as metatranscriptomic by JGI Gold (Accession no.: Gs0128947). *G. huxleyi* (formerly *Emiliania huxleyi*) (CCMP1516) gene models downloaded as peptide sequences (v.1.0 best proteins; Read *et al*., 2013) were searched against the UniProt Swiss‐Prot peptides with blastp and submitted to BlastKoala v.2.2 (Kanehisa *et al*., 2016) for KO number annotations. Peptide sequences of both *S*. *apsteinii* and *G. huxleyi* were considered further if they were matched with a primary KO and blastp hits with e‐values < 1e^−5^.

Further curation of hit sequences focused on KO and homologs acting in KEGG Pathways characterized within cellular processes: transport and catabolism. These pathways were phagosome (04145), endocytosis (04144), lysosome (04142), autophagy (animal – 04140, yeast – 04138, and other – 04136), and mitophagy (animal – 04137, yeast – 04139). Results from blastp and GhostKoala were queried against pathway associated KO numbers using SQLite (v.3.22.0) and DB Browser for SQLite (v.3.11.2). Transcript IDs of *S. apsteinii* that are associated with each pathway are in Tables S1–S4.

## Results

### Phagocytosis and identification of the specialized acidic vacuole

**Fig. 1:**
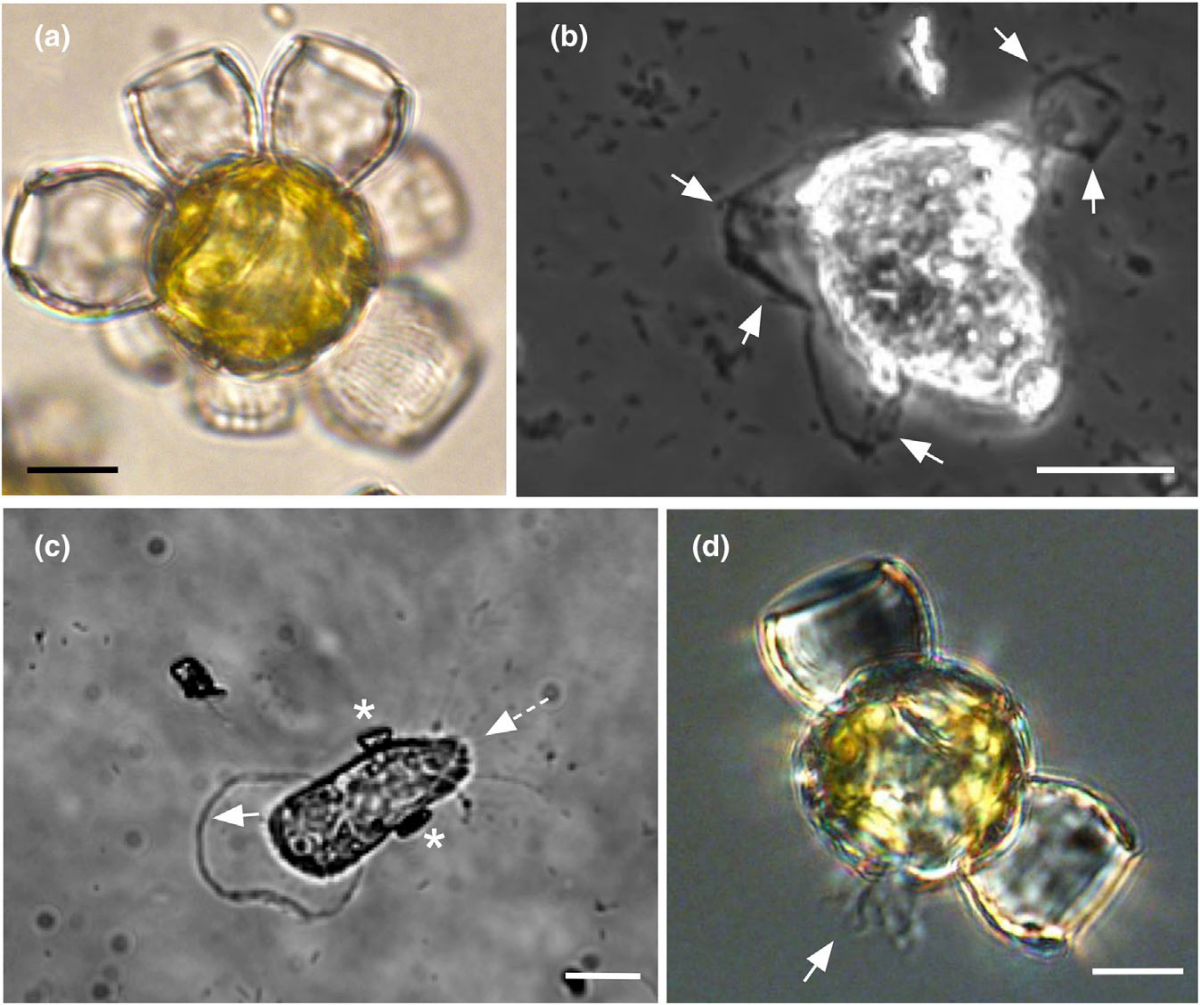
Amoeboid behavior and utilization of lamellipodia and filopodia to contact prey particles by *Scyphosphaera apsteinii*. (a) Brightfield color micrograph of a calcified *S. apsteinii* with a coccosphere comprising large barrel‐like lopadoliths and flat plate‐like muroliths. (b) Phase contrast black and white micrograph of a decalcified cell extending lamellipodia with characteristic thickened margins along the surface of a coverslip. Lamellipodia interact with bacteria and particulate matter (arrows). See Supporting Information Video S1. (c) Phase contrast black and white micrograph of a decalcified cell bearing two recently secreted coccoliths (white asterisk) actively moving across a coverslip (arrows indicate direction of movement). (d) Color differential interference contrast (DIC) micrograph of a calcified cell showing lamellipodia and filopodia (arrow) emerging from the cell through the coccosphere to the cell exterior. See Video S2. Bars, 10 μm.

*Scyphosphaera apsteinii* is heavily calcified and produces plate‐like muroliths and urn‐shaped lopadoliths in its native form (Fig. 1a). Naked cells were observed producing lobed lamellipodia that interacted with bacteria and other particles on the surface of the coverslip dish (Fig. 1b,c; Video S1) when cells were decalcified to follow the progression of calcification. Protrusion of slender filopodia from between coccoliths of normally calcified cells (Fig. 1d) suggests a natural mechanism for prey capture and uptake (Video S2). These observations indicated that this coccolithophore species could actively engulf food particles and, thus, was mixotrophic. We designed a series of experiments to definitively test for phagotrophy by *S. apsteinii* and characterize the mechanism of ingestion and processing.

**Fig. 2:**
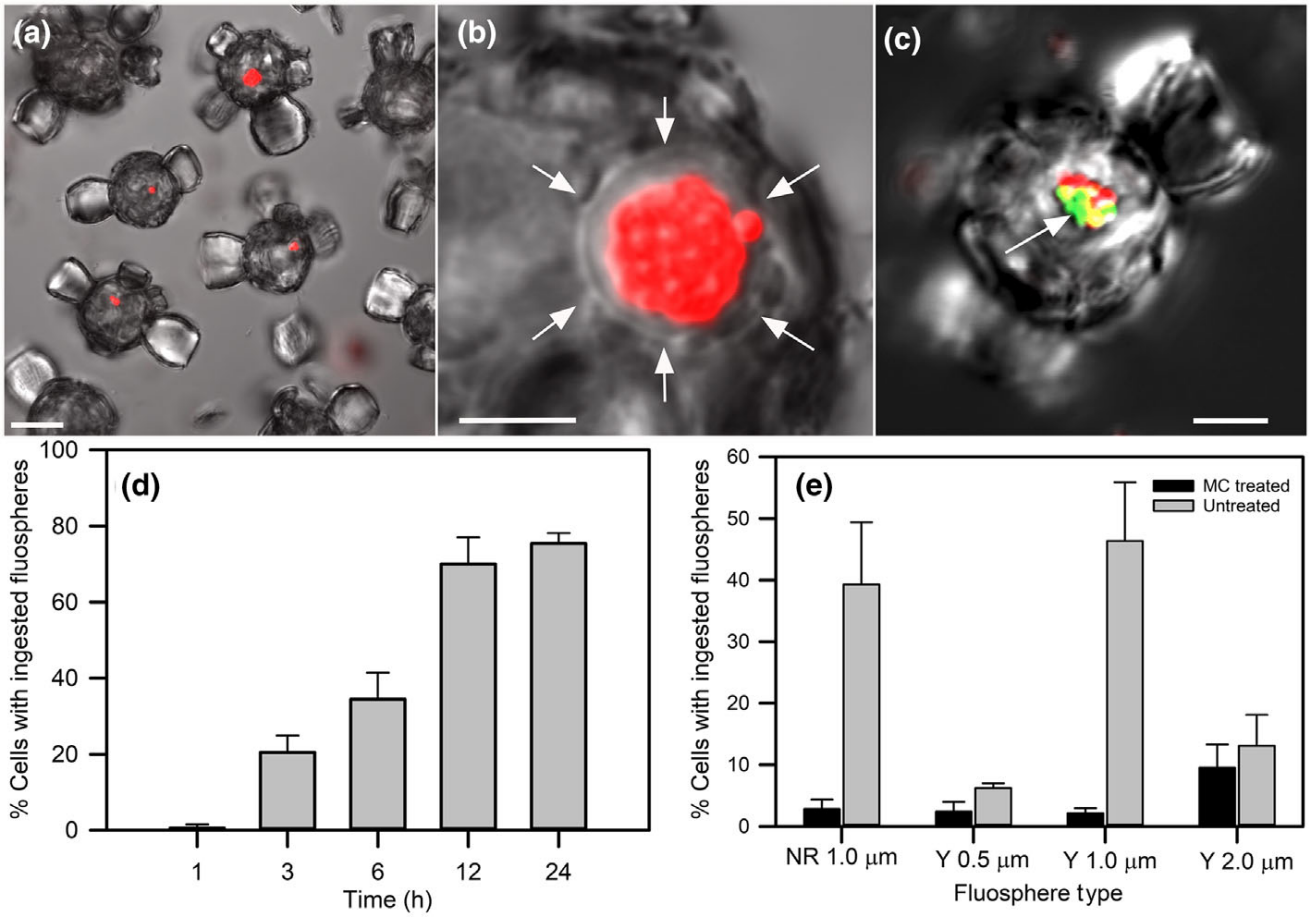
Phagotrophy in the coccolithophore *Scyphosphaera apsteinii*. FluoSphere beads localized to a single vacuole, while ingestion was both size selective and dependent on surface treatment. (a) Confocal section overlay of differential interference contrast (DIC) and red fluorescence of multiple cells with ingested untreated (carboxylated) 1 μm Nile Red (NR) FluoSphere beads after 12 h incubation (bar, 10 μm). (b) Detail of confocal section of a cell that has ingested > 20 NR beads that are within a vacuole bound by a single membrane (white arrows; bar, 5 μm). (c) Ingested 1 μm NR and Yellow (Y, pseudocolored green). FluoSphere beads co‐locate to the same single vacuole (arrow) for processing. A colocalization signal (yellow) is seen where the two types of beads are in close proximity within the vacuole. Bar, 10 μm. (d) Time course of prey uptake (1 μm NR Beads) over 24 h. By 12 h 70.0% cells have ingested beads and at 24 h 75.4% of cells ingested one or more bead (*n* > 10 per time point over 3 independent experiments, error bars = SE). (e) *Scyphosphaera apsteinii* cells exhibited preference for size and surface properties for bead ingestion: 1 μm over 0.5 or 2.0 μm, and untreated (carboxylate‐modified, negatively charged) over methylcellulose (MC) treated (neutrally charged), respectively. Each bar represents the average (±SE) of *n* > 100 cells scored per treatment for 3 independent experiments. There was no significant difference in ingestion between 1 μm untreated NR and Y beads (one‐way ANOVA, *F* = 0.256, *P* = 0.639). Uptake rates of 1 μm untreated beads were significantly greater than those of both 0.5 μm and 2.0 μm untreated Y beads (*F* = 11.87, *P* = 0.008). Considering the preferred prey size of 1.0 μm, there was a significant decrease in ingestion when FluoSphere beads were MC treated for both 1.0 μm Y (*F* = 21.41, *P* = 0.010) and 1.0 μm NR (*F* = 12.67, *P* = 0.024).

*Scyphosphaera apsteinii* was tested for phagocytosis by exposing them to fluorescently labeled beads. All ‘untreated’ beads were carboxylated and negatively charged. The majority of *S. apsteinii* cells ingested the untreated 1 μm beads (Fig. 2a), which localized to a single membrane‐bound compartment (Fig. 2b,c). Within 3 h, cells ingested one or more beads and by 12 and 24 h, 70.0 ± 7.0% and 75.4 ± 2.8% of the cells, respectively, had ingested and accumulated beads (Fig. 2d). Size and surface properties of the beads significantly influenced the degree to which they were ingested by *S. apsteinii*. The untreated 1.0 μm beads were ingested at the highest frequencies with 40–45% of cells containing beads after 10 h of exposure. Smaller (0.5 μm) and larger (2.0 μm) untreated beads resulted in significantly lower uptake; only 5–10% of cells actively ingested them. Treatment of 1 μm beads with methylcellulose, which created a neutrally charged bead surface, significantly suppressed ingestion of beads of all sizes and the frequency of cells ingesting them declined to < 5% (Fig. 2e). Two additional species, *G. huxleyi* and *Coccolithus braarudii*, were tested. No ingestion of treated or untreated beads was observed in either species (Table S5).

**Fig. 3:**
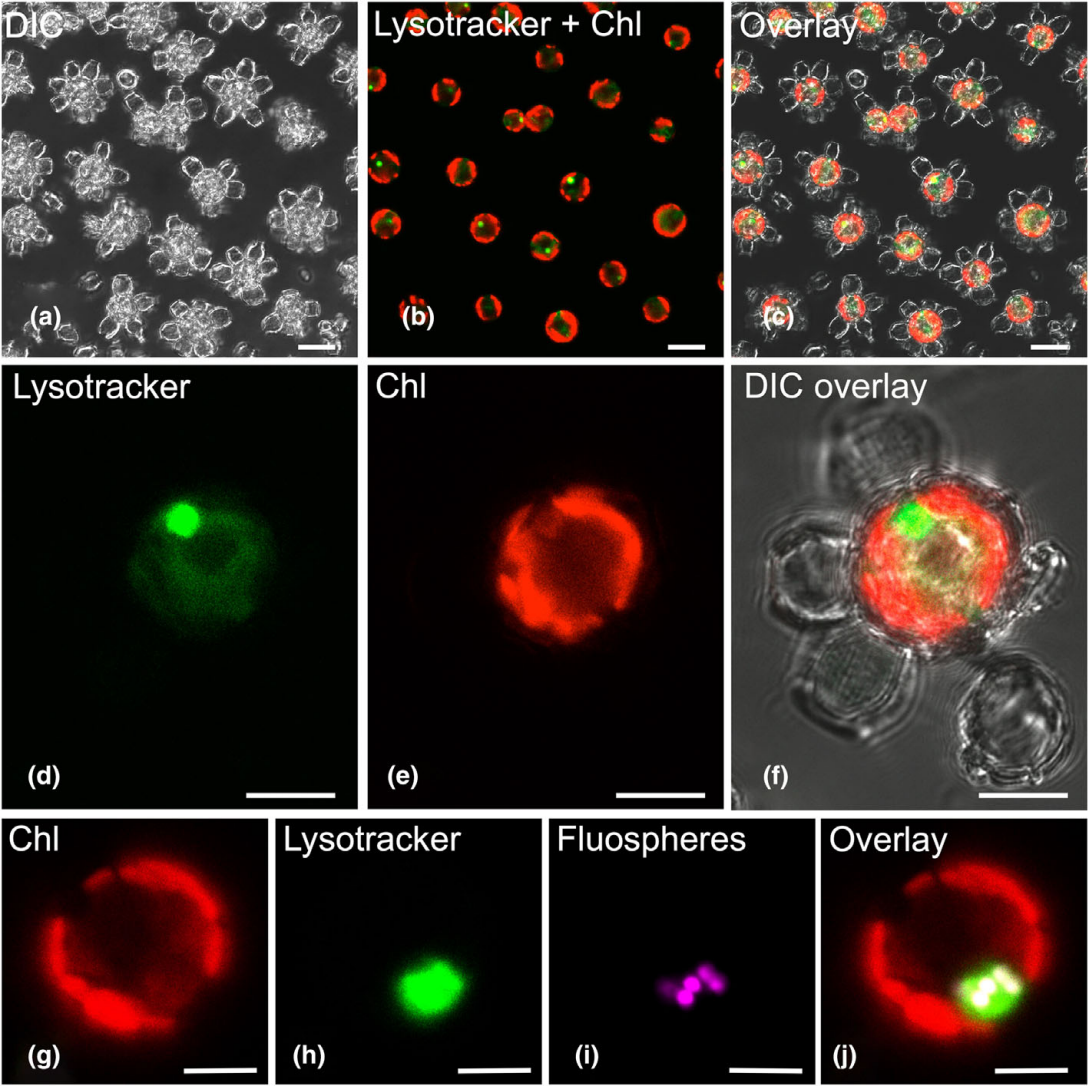
A single prominent acidic vacuole colocalizes with ingested FluoSphere beads in *Scyphosphaera apsteinii*. (a–c) Single confocal section from the left: differential interference contrast (DIC) (grayscale), Lysotracker Green (LTG) and Chl (red), and overlay of all channels. (d–f) Confocal section detailing a single cell with a LTG‐stained vacuole. (g–j) Representative single confocal section through a cell that was incubated for 4 h with 1 μm Y beads (pseudocolored magenta) and subsequently stained with Lysotracker Red (LTR). Colocalization of the beads and LTR signal is evident in the overlay (beads appear white). Images collected with laser confocal microscopy. Bars: (a–c) 20 μm; (d–j) 10 μm.

To determine if the vacuoles were acidic and potentially digestive in nature, cells were stained with the acidotropic fluorescent probe Lysotracker Green (LTG). A single prominent LTG‐stained vacuole 3.02 μm in diameter (±1.03 μm, *n* = 16 cells measured from 4 replicate cultures) was present in up to 100% of cells (Fig. 3a–f). Incubation of cells with fluorescently labeled beads followed by LTG staining demonstrated that the ingested beads colocalized with and accumulated in the acidic vacuole in the majority of cells (*c*. 70%; Fig. 3g–j; Table S6), indicating that ingested particles were trafficked and processed in a single specialized acidic vacuole.

### Distinction between the acidic vacuole and polyP‐containing vacuole

**Fig. 4:**
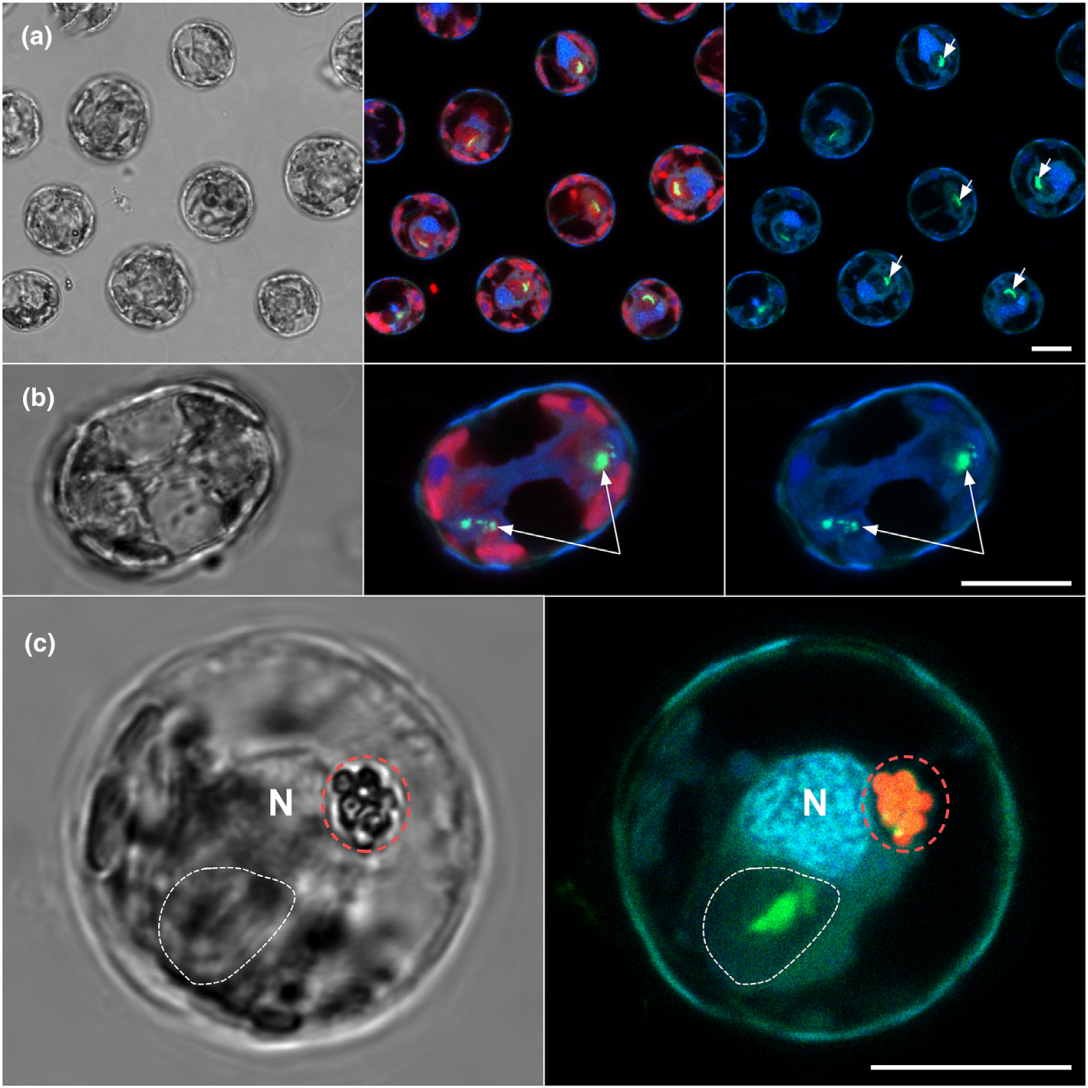
The acidic vacuole is distinct from the polyphosphate (polyP) storage compartment of *Scyphosphaera apsteinii*. (a) Left: confocal differential interference contrast (DIC) micrograph of *S. apsteinii* cells (glutaraldehyde‐fixed and decalcified). Middle: overlay of DAPI (blue), polyP (green) and Chl (red) signals. Right: DAPI/ polyP signals only. Most cells exhibit an intense polyP signal in a discrete large vacuole‐like compartment adjacent to the nucleus (examples indicated by white arrowheads). (b) The coccolithophore polyP storage organelle divides (white arrows) and is present in daughter cells undergoing cytokinesis. Left: confocal DIC micrograph of a decalcified dividing *S. apsteinii* cell. Middle: overlay of DAPI (blue), polyP (green) and Chl (red) signals. Right: DAPI/polyP signals only. (c) The single primary polyP store and acidic vacuole are spatially distinct. Left: confocal DIC micrograph showing position of polyP compartment (white dotted line), nucleus (N) and ingested beads in the digestive vacuole (red dotted circle). Right: corresponding confocal fluorescence micrograph indicating position of polyP compartment (green), nucleus (blue) and NR beads (red). Bars, 10 μm.

The presence of only one prominent acidic vacuole led us to hypothesize that it may represent the same subcellular structure as the calcium and polyP‐rich body found in *G. huxleyi* (Sviben *et al*., 2016; Gal *et al*., 2017) and eukaryotic acidocalcisomes more broadly. Most of the cells stained with DAPI and imaged with dual emission contained large vacuoles with green fluorescence indicative of polyP stores (Fig. 4a). PolyP‐containing vacuoles were observed at both poles of actively dividing cells (Fig. 4b). Dual labeling with 1 μm fluorescently labeled beads and DAPI revealed that the acidic bead‐containing vacuole and compartments containing polyP were physically separate and therefore functionally distinct from one another (Fig. 4c).

### Ingestion of bacteria and cyanobacteria

**Fig. 5:**
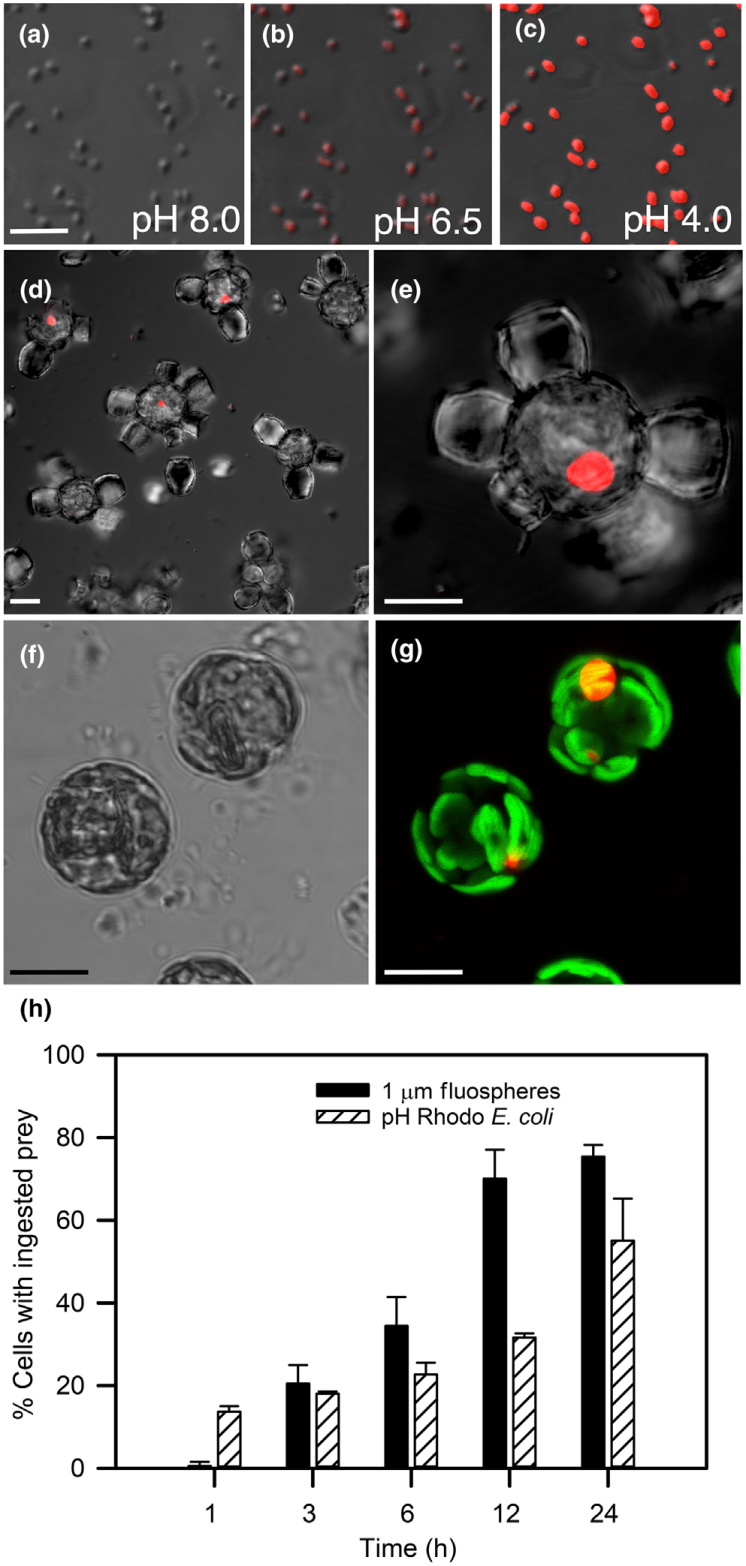
Bacteria are phagocytosed by the coccolithophore *Scyphosphaera apsteinii* and processed in a single prominent acidic vacuole. (a–c) Confocal differential interference contrast and Rhodamine channel overlay images of pHrodo *Escherichia coli* BioParticles with seawater of varying pH perfused through the coverslip chamber. Cells exhibit a strong red fluorescence at pH < 5.0. (d, e) Ingested pHrodo *E. coli* are processed within and indicate the single dominant acidic vacuole (pH < 5.0) in *S. apsteinii* cells. (f) Brightfield confocal image of two decalcified cells and (g) confocal maximum projection of a Z‐stack demonstrating location of pHrodo *E. coli*‐labeled vacuoles (red) and chloroplasts (green). (h) Representative time‐course experiment using pHrodo *E. coli* as prey. Frequency of cells with ingested prey (% ± SD, *n* = 3 experimental replicates) were determined from a minimum of 120 cells analyzed per replicate per timepoint. The nile red fluosphere bead uptake time course is the same data as in Fig. 2. The time course for pHrodo *E. coli* uptake followed a similar pattern to 1 μm nile red bead uptake. Bars: (a–c) 5 μm; (d–g) 10 μm.

We confirmed that biologically and ecologically relevant particles were ingested and localized to the same specialized acidic vacuole as the fluorescence beads by incubating *S. apsteinii* with the pH indicating bacterium pHrodo *E. coli* BioParticles. pHrodo *E. coli* are nonfluorescent in the extracellular seawater pH of 8.0 but fluoresce brightly at pH < 5 (Fig. 5a–c). The pHrodo *E. coli* were ingested within minutes and trafficked to the acidic vacuole for processing and detected as an aggregated red fluorescent signal in up to 60% of the exposed S. *apsteinii* cells by 24 h (Fig. 5d–g). The time course for ingestion of pH Rhodo *E. coli* followed a similar pattern to that of 1 μm beads (Fig. 5h). Co‐incubation experiments revealed colocalization of ingested pHrodo *E. coli* signal and fluorescently labeled beads, further demonstrating the central role of the acidic vacuole in processing all ingested particles (Fig. S2b–h).

**Fig. 6:**
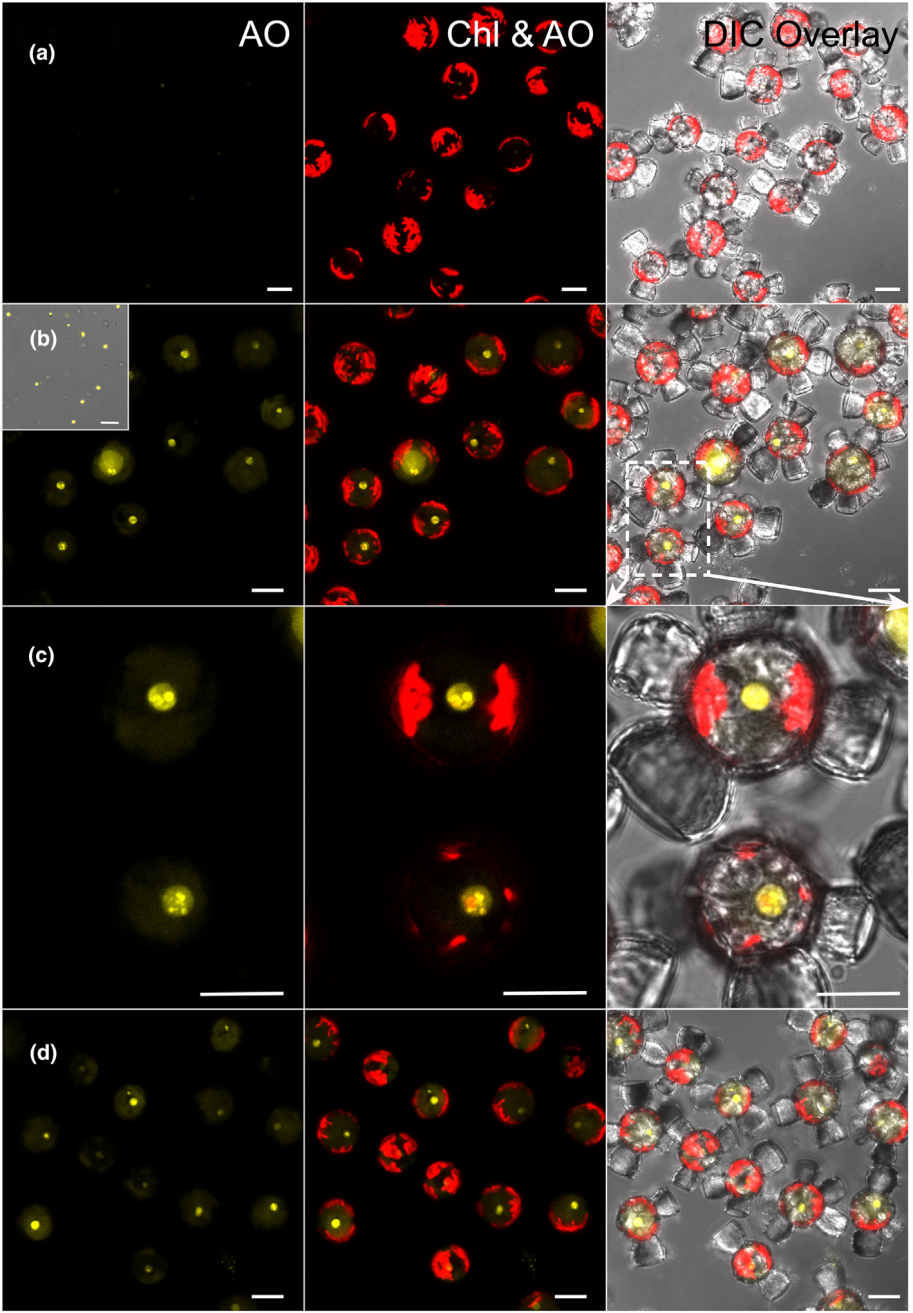
Phagotrophy of marine bacteria by *Scyphosphaera apsteinii*. For each panel: left, confocal images of fluorescently labeled bacteria (Acridine Orange (AO), yellow); middle, Chl (red) and bacteria (AO) signals; and right, DIC overlay with the two fluorescent channels. (a) Control cells that are unfed. (b) Uptake of AO stained *Synechococcus* sp. (inset). After 12 h the majority of *S. apsteinii* have engulfed bacteria. (c) Detail of two cells from panel (b) as indicated by white dotted region of interest. The centrally located vacuole is clearly visible as Acridine Orange dye from the *Synechococcus* sp. prey diffuses through the organelle. Individual AO‐labeled bacterial cells in the vacuole give rise to intense punctate labeling. (d) Uptake and digestion of AO‐labeled heterotrophic marine bacteria. *S. apsteinii* cells were examined 12 h after incubation with prey. Bars: (a, c, d) 10 μm; (b) 5 μm.

Marine bacteria were also tested as a prey source by fluorescently labeling *Synechococcus* sp. (cyanobacterium) and an assemblage of marine heterotrophic bacteria that were batch‐cultured from an aliquot of the nonaxenic *S. apsteinii*. In separate experiments, 40–60% of *S. apsteinii* cells contained ingested Acridine Orange‐stained, *Synechococcus* sp. or heterotrophic bacterial cells at 12 h (Fig. 6).

### Constitutive expression of the acidic vacuole and chlorophagy

**Fig. 7:**
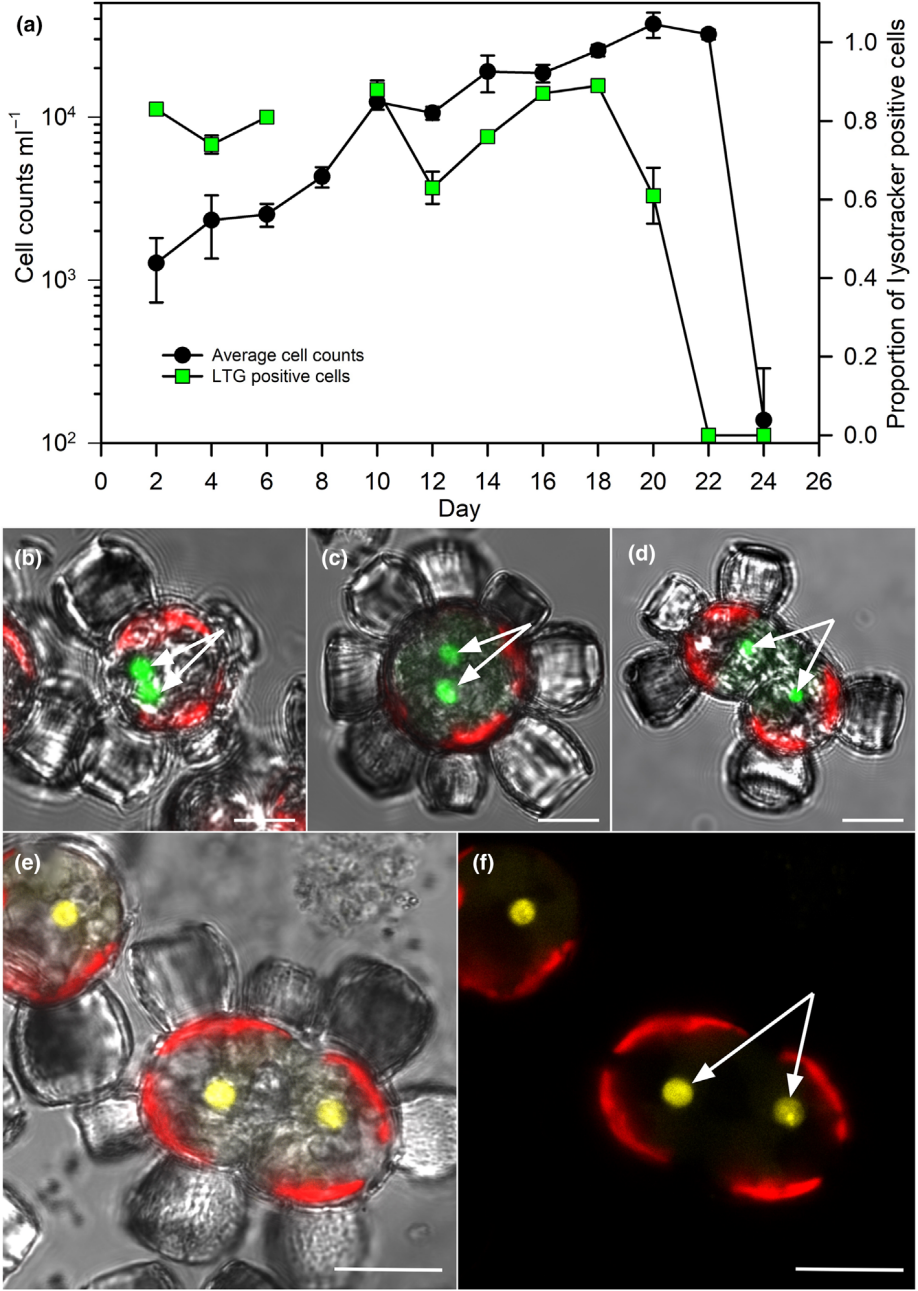
The central acidic vacuole of *Scyphosphaera apsteinii* is a constitutive organelle. (a) The majority of cells possess a detectable Lysotracker Green (LTG)‐stained vacuole (green squares) as cell number (black circles) increases through early and exponential growth phases of a batch culture (normalized % LTG positive cells ± SD, *n* = 3 experimental replicates). Due to unforeseen circumstances, we were unable to complete the LTG analysis on day 8. A similar pattern was observed in a second experiment (Supporting Information Fig. S3). (b–d) Confocal sections with LTG (green), chlorophyll (red) and differential interference contrast overlay in which the acidic LTG‐stained organelle (white arrows) has divided before cytokinesis. (e, f) *Scyphosphaera apsteinii* cells fed Acridine Orange‐labeled heterotrophic bacteria (yellow) that are localized to the acidic vacuole (while arrows) that has undergone division before cytokinesis. Bars, 10 μm.

We next examined whether the acidic vacuole was transient or constitutive. The presence of the vacuole was monitored over the course of the *S. apsteinii* growth curve using LTG staining combined with flow cytometry (Figs 7a, S3). The proportion of cells with an acidic vacuole determined using this high‐throughput approach was comparable to that determined by manually scoring cells visualized with confocal microscopy (Fig. S1a–d,h). During exponential growth, between 60% and 90% of *S. apsteinii* contained a prominent LTG‐stained vacuole, but a dramatic loss of LTG‐stained vacuoles preceded the population decline by 3 d, suggesting an increase in pH (i.e. loss of LTG signal) and shift in the biochemical nature of the vacuole that corresponds to senescence (Fig. 7a). Cells in early and late stages of cytokinesis were observed to contain two Lysotracker stained vacuoles, one destined for each presumptive daughter cell (Fig. 7b–d). Similarly, vacuoles containing remains of fluorescently labeled bacteria divided before cytokinesis (Fig. 7e,f). In a further independent experiment, the quantum yield of PSII remained stable as cells entered the late‐exponential phases of growth, but the proportion of cells containing a stained vacuole declined sharply to less than 40% (Fig. S3).

**Fig. 8:**
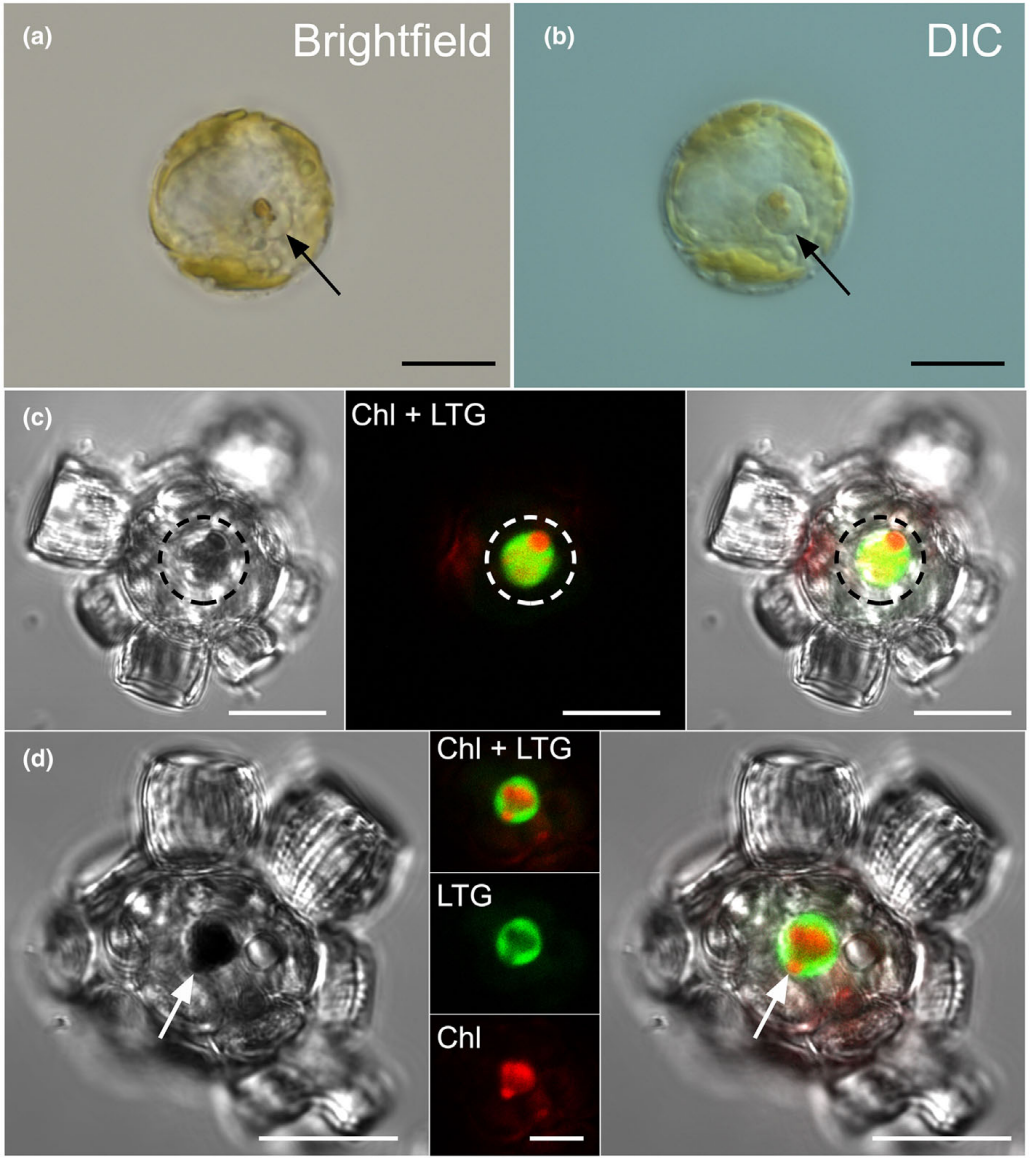
The digestive vacuole of *Scyphosphaera apsteinii* plays a central role in chlorophagy. (a, b) Representative brightfield and differential interference contrast (DIC) images of a decalcified cell with a vacuole containing pigmented chloroplast fragments. Supporting Information Video S3 shows the corresponding Z‐stack through this cell. (c) Left confocal DIC: *S. apsteinii* cell with prominent vacuole with dense material within (black dotted region) that corresponds to chloroplast fragments. Middle confocal section: Lysotracker Green (LTG) and chlorophyll (Chl) (red) signals are closely associated. Right overlay: red Chl signal corresponds to the dense material within the vacuole. (d) Additional example showing dense material that corresponds to Chl within the acidic vacuole left to right: DIC, confocal sections of Chl and LTG, and overlay of all signals. The fluorescent channels demonstrate the physical colocalization of the chloroplast fragment and LTG in this vacuole, but the LTG is excluded from the chloroplast fragment. Bars: (a–c) 10 μm; (d) (right and left) 10 μm, (middle) 5 μm. See Fig. S4 including Videos S4 and S5 for additional data.

Significantly, and unexpectedly, we often observed a physical colocalization of pigmented and autofluorescent structures in mid‐exponential and early stationary‐phase cells, indicating Chl surrounded by the LTG signal of the prominent acidic vacuole (Fig. 8; Video S3). Imaging optical sections through Lysotracker red‐stained cells (in z‐stacks) confirmed that Chl fluorescence from the vacuoles was distinct from intact chloroplasts (Fig. S4; Videos S4, S5). Chlorophagic vacuoles were detected in 34.0% of cells (±10.0, *n* = 3 replicate experiments with an average of 148 cells scored for each; Table S7). These vacuoles were likely in the early stages of chlorophagy in which chloroplast fragments (Fig. 8a,b), recently transported to the acidic vacuole via an autophagic pathway, were not yet fully degraded. These chlorophagic vacuoles characteristically excluded the LTG from the plastid fragment (Fig. 8c, d), suggesting the plastid membrane was not yet permeable and proteolytic degradation had not commenced.

### Ultrastructure of the single prominent acidic vacuole

**Fig. 9:**
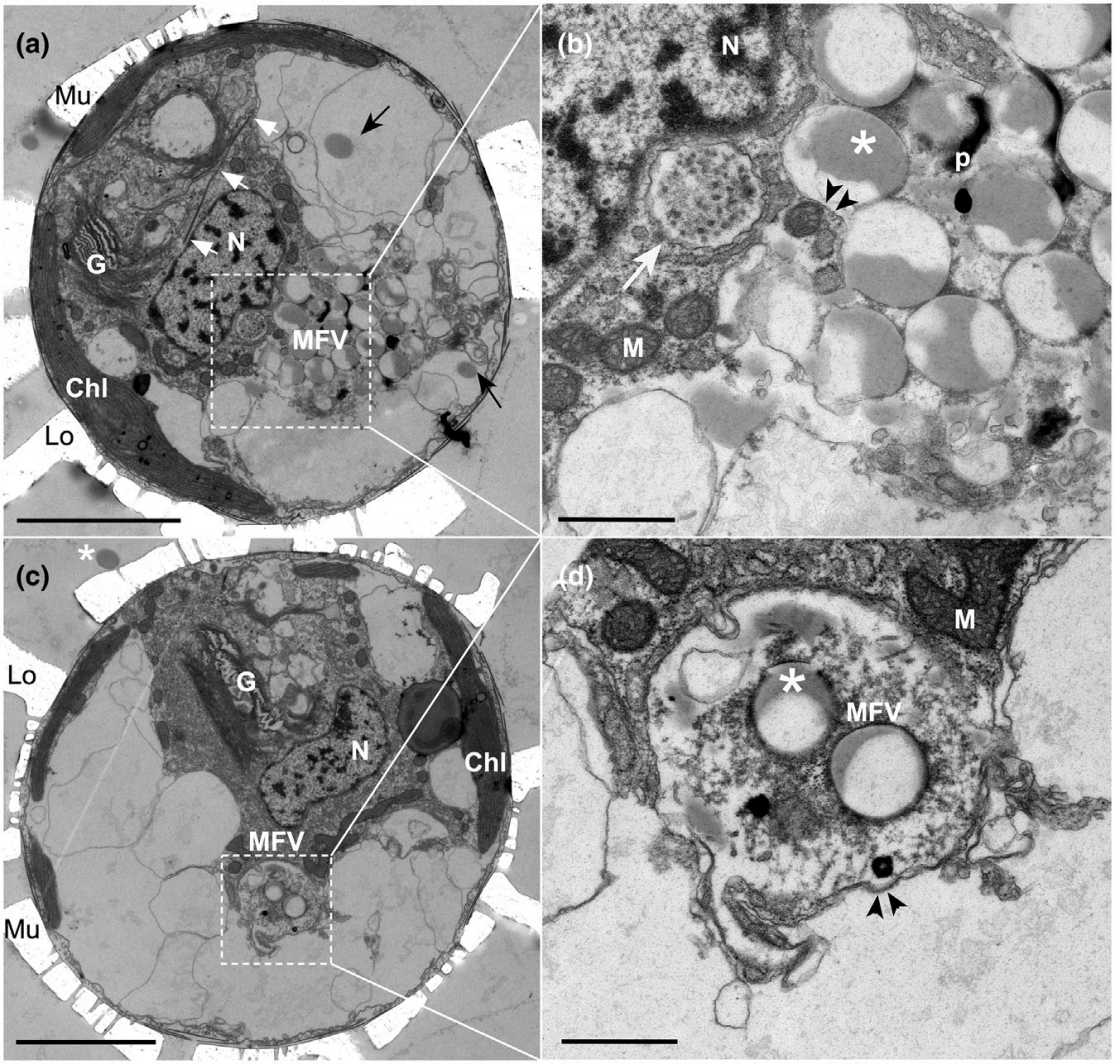
Ultrastructure of the multifunctional vacuole (MFV) of *Scyphosphaera apsteinii*. (a) TEM micrograph of whole *S. apsteinii* with ingested 1 μm FluoSphere beads tightly packed and undergoing degradation in the MFV adjacent to the nucleus (N). Intact beads presumably in transit to the MFV (black arrows), newly synthesized baseplate (white arrows) in a Golgi body‐derived vesicle, Golgi body (G), chloroplasts (Chl). Extracellular coccoliths (Mu = murolith, Lo = Lopadolith) represented by holes in the resin left by the dissolved calcite. Bar, 5 μm. (b) Detail of (a): The MFV is bound by a single membrane (black arrowheads) and is closely associated with the nucleus (N). The 1 μm beads (white asterisk) are in a matrix of darkly stained amorphous material (p), likely a suite of enzymes and other proteins related to degradation. A putative autophagic vesicle (white arrow) may be in transit to the MFV. Mitochondria (M) in the adjacent cytoplasm. (c, d) Whole cell TEM micrograph and detail of membrane‐bound (black arrowheads) MFV containing two beads (white asterisk) that are surrounded by densely stained material and have undergone significant degradation. Note in (c) the intact undigested bead on outside of the cell (white asterisk). Bars: 10 μm (a, c); 1 μm (b, d).

Four independent cultures of *S. apsteinii* were incubated with 1 μm fluorescently labeled beads to verify active ingestion and aid in identifying the acidic vesicle for ultrastructural analysis with TEM. In all replicates, the bead‐containing vacuole (Fig. 9) was consistently located at the edge of the cytoplasmic core adjacent to the nucleus and between the cytoplasmic core and the periphery of the cell and chloroplasts, which are closely associated with the plasma membrane. The remainder of the cell was filled by multiple larger vacuoles that did not have discernable intra‐vacuolar structure; however, these vacuoles were observed to contain intact beads presumably in transit to the acidic vacuole for processing (Fig. 9a). The digestive bead‐containing vacuole, which frequently contained small vesicles of disorganized tubular structure, was bound by a single membrane (Fig. 9). Dark, electron dense material was observed around and between beads located within the vacuole and some beads were degraded with irregular shape, no longer exhibiting the smooth perimeter of beads found within the peripheral vacuoles or outside the cells (Figs 9, S5). The digestive vacuole often contained > 10 beads in cross section (and in some cases a great deal more) resulting in an approximate vacuole diameter of > 5 μm. Four independent cultures that were not exposed to beads were also analyzed using TEM to identify the endogenous acidic digestive vacuole. We observed many examples of a membrane‐bound vacuole adjacent to the nucleus in the cytoplasmic core. These vacuoles were *c*. 3 μm across and contained a range of dense punctate material, vesicles, and other organic matter (Fig. S6). While the absence of tracer beads makes these vacuoles difficult to identify with certainty, the location, size, and features including densely stained organic material in various forms strongly suggest these are the native state acidic digestive vacuole.

### Genetically based conceptual model

**Fig. 10:**
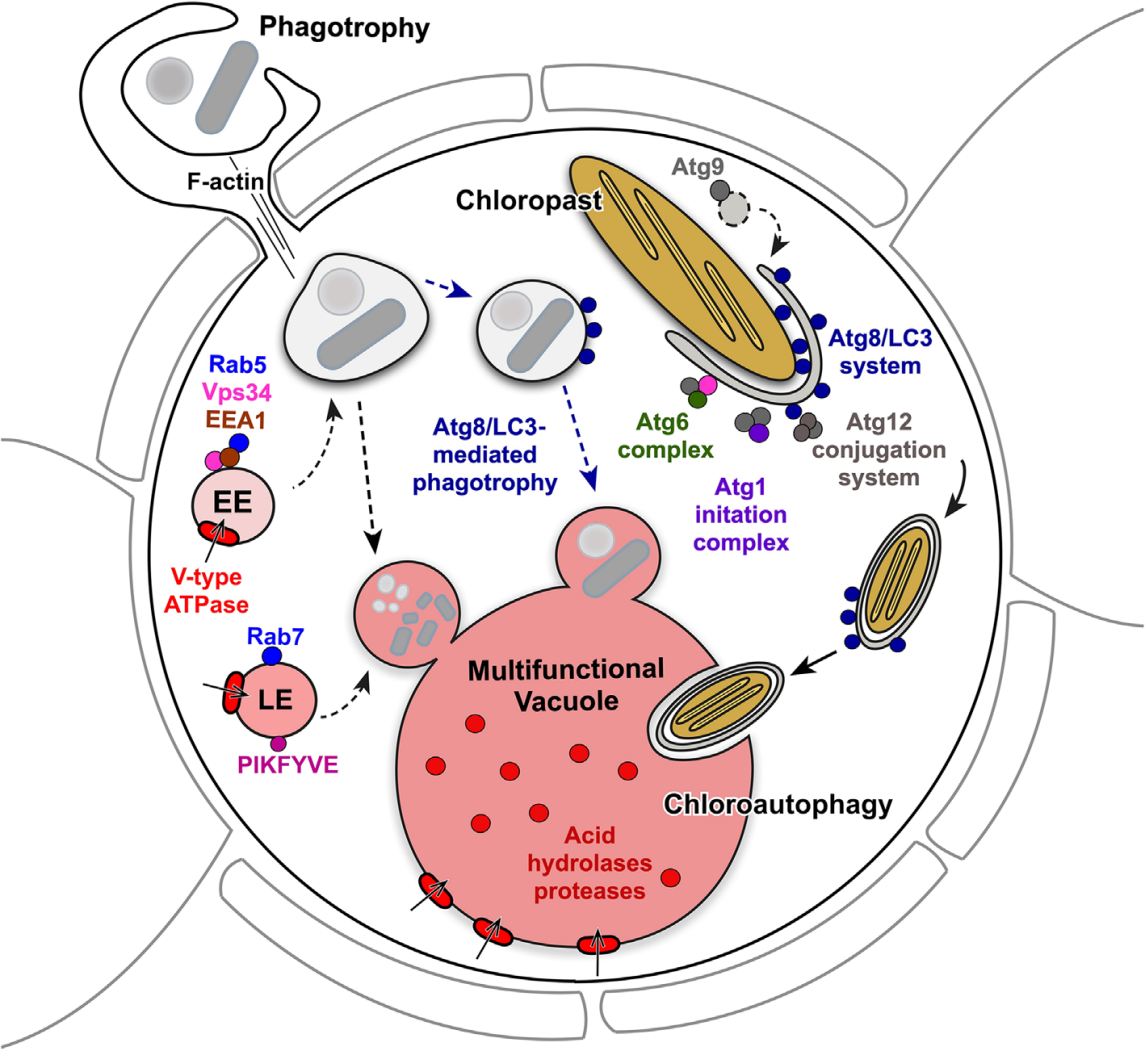
Conceptual model of the multifunctional vacuole (MFV) and its role in phagocytosis and autophagy in coccolithophores. Core proteins from *Scyphosphaera apsteinii* are represented by colored dots, while those identified only in *Gephyrocapsa huxleyi* are gray; protein identification was made from translated transcripts and gene models, respectively. Two potential pathways targeting the initial phagocytic vesicle to the digestive vacuole are indicated by dashed arrows. The endocytic pathway contains early endosomes (EE) and late endosomes (LE). The hypothesized chloro‐autophagic pathway targeting chloroplast fragments to the MVF is indicated with solid black arrows. The core Atg1 (purple) complex includes Atg13 (gray) and Atg101 (gray). The core Atg6 (green) complex includes Vps34 (pink) and Vps15 (gray).

Given the single constitutive acidic vacuole in which ingested prey and chloroplast fragments (but not polyP) were localized, we investigated the genetic basis of phagocytosis and autophagy in *S. apsteinii* and the model coccolithophore *G. huxleyi* by comparing annotated transcripts and gene models, respectively, with the genes in KEGG reference pathways for the phagosomes, endocytosis, autophagy, and lysosomes (Tables S8–S11). We identified transcripts and gene models encoding core sets of proteins in *S. apsteinii* and *G. huxleyi* that could control phagocytosis from prey sensing to degradation through a classical endocytosis pathway or by tagging the phagocytic vesicle with an ATG8/LC3 (ubiquitin‐like) homolog, thus targeting it to the acidic vacuole (Fig. 10; Tables S8–S11). Homologs encoding the major proteins and protein complexes controlling autophagy were also identified, confirming the genetic means for chlorophagy in the coccolithophores (Table S11). We propose a conceptual model of these pathways that is supported by both our experimental results and genetic evidence for the proteins that control them (Fig. 10). We hypothesize that the acidic vacuole coordinates phagotrophy and autophagy, including chlorophagy, and that intermediate vesicles containing cargo destined to be degraded and recycled are targeted to this centralized structure where the two pathways converge.

## Discussion

We present unequivocal evidence of phagotrophy and prey processing within a single prominent acidic vacuole in the constitutively photosynthetic, nonmotile, and heterococcolith‐bearing coccolithophore *S. apsteinii*, demonstrating that this species is a constitutive mixotroph (Mitra *et al*., 2016; Flynn *et al*., 2019). Prey ingestion was selective, and we hypothesize that it was mediated by filopods extending from between noninterlocking coccoliths. The acidic vacuole to which prey were targeted was conserved during cell division and expressed throughout the cell cycle and into the early stationary phase of growth. Chloroplast fragments were targeted to the same acidic vacuole as the prey. This organelle is therefore multifunctional and plays a unique and central role in phagotrophy and autophagy, pathways for which core regulatory genes were identified. This multifunctional vacuole (MFV) is distinct from a larger vacuole containing polyP stores.

Phagocytosis in *S. apsteinii* occurs through both novel and conserved structures and pathways. The noninterlocking structure of the *S. apsteinii* coccosphere provides a means for the cell to interact with prey using filopodia that extend from between the coccoliths. Given that numerous species within the Zygodiscales also produce noninterlocking coccospheres, the coccolithophore contribution to mixotrophy is currently underestimated. Prey uptake by *S. apsteinii* was sensitive to the surface properties (e.g. charge) and size of prey particles. While the mechanisms of prey uptake in coccolithophores requires further examination, prey recognition likely occurs using carbohydrate‐binding lectin proteins such as mannose receptors as has been demonstrated in the marine protist *Oxyrrhis marina* (Wootton *et al*., 2007). Interestingly, transcripts for C‐type (Ca^2+^‐dependent) mannose receptors were identified in *S. apsteinii*. Thus far, there are two described routes for phagosomal processing, a conserved endocytic pathway and tagging with ubiquitin‐like autophagy homologs of Atg8/LC3 that would enable acidification (Picazarri *et al*., 2015) and transport to the lysosome‐like MFV. The exact mechanism by which ingested prey particles are translocated to the MFV of *S. apsteinii* is unclear, in part because beads were observed within large peripheral vacuoles that were largely empty of identifiable ultrastructure. Modification of the phagosomal or vacuolar membrane and lumen are supported by the presence of transcripts and gene models encoding enzymes for phosphotidyl inositol signaling, the V‐ATPase complex to acidify the compartment, and acid hydrolases for degradation of prey.

Interestingly, we observed significant deformation and loss of the polystyrene FluoSphere beads in the MFV that was not observed in beads transiting the peripheral vacuoles or outside the cells. The pattern of resin and electron‐dense material around the beads within the MFV suggests that they were degraded by digestive enzymes (Stock *et al*., 2020). While nano‐ and microplastics tend to affect phytoplankton and their grazers negatively (Kögel *et al*., 2020), reported effects are generally due to extracellular exposure and adsorption (Chae *et al*., 2018). Our observations suggest that intracellular degradation of ingested plastics such as polystyrene beads by mixoplankton may be underestimated and could significantly influence the trophic transfer of microplastics through transformation of these particles to dissolved organic compounds.

In a first observation for phytoplankton, we describe chlorophagy that was readily detected when *S. apsteinii* had reached the early stationary phase of growth. Nonchlorophagic autophagy has been described in *G. huxleyi* (Schatz *et al*., 2014; Shemi *et al*., 2015) and is implicated in phosphate limitation (Shemi *et al*., 2016), with notable differences from our observations of chlorophagy. The single membrane‐bound MFV of *S. apsteinii* frequently contained smaller degradative vesicles and was expressed constitutively, whereas the likely autophagic vesicles of *G. huxleyi* were bound by double membranes and formed when cells were phosphate‐limited (Shemi *et al*., 2016). Across the eukaryotes, autophagy is regulated by the dynamics and requirements of growth. Autophagy recycles aggregated proteins and damaged or surplus organelles and cellular machinery using distinct mechanisms in response to environmental and pathological stress (Morishita & Mizushima, 2019). Given the discrete shape and small size of the Chl fluorescence in the MFV relative to the size of the large lobed chloroplasts, we hypothesize that chlorophagy in *S. aptsteinii* occurred through a microphagic process similar to that in plants where ATG proteins tag and coordinate pruning of damaged chloroplasts and coordinate transport of the resulting fragments to the vacuole (Nakamura *et al*., 2018). We propose that chloroplast fragments are transported into the MFV through invagination of its membrane because the MFV and smaller degradative vesicles were bound by a single membrane instead of the double membranes that characterize macrophagy (Masclaux‐Daubresse *et al*., 2017). Given the relatively high number of chlorophagic cells detected, it seems likely that chlorophagy is critical in maintaining plastid health and resisting oxidative stress in phytoplankton, as has been demonstrated for higher plants (Izumi *et al*., 2017; Miyagishima, 2023).

In *S. apsteinii*, as well as motile mixotrophic haptophytes, ingested particles are targeted to a single vacuole, unlike most unicellular heterotrophs and mixotrophs that contain multiple food vacuoles that progress individually through the digestion process (Ryter & de Chastellier, 1977; Carvalho & Granéli, 2006; Frail‐Gauthier *et al*., 2019). Interestingly, *Ancoracysta twista*, the heterotrophic single‐species sister taxon to the haptophytes, appears to have only one prominent food vacuole (Janouškovec *et al*., 2017). Plants and yeast contain a single centralized vacuole, that is the target for autophagic vesicles and multivesicular bodies, where both degradation of recycled cellular materials and nutrient storage, especially of cations and phosphates, occurs (Yang *et al*., 2017).

In many eukaryotes, separate acidic organelles, acidocalcisomes, store cations (namely Ca^2+^) and polyphosphates (Goodenough *et al*., 2019). In trypanosomes, acidocalcisomes play an integral role in autophagy (Li & He, 2014). Recently, a large vacuole containing a calcium rich polyP body that supplies calcium for coccolith production was identified in *G. huxleyi* (Sviben *et al*., 2016; Gal *et al*., 2017). Similarities between the calcium‐polyP bodies of *G. huxleyi* and *Chlamydomonas reinhardtii* led to the conclusion that the vacuole in *G. huxleyi* functions as an acidocalcisome (Gal *et al*., 2018). Given the prominence of the acidic MFV in *S. apsteinii*, we hypothesized that it too stores calcium and polyP, however, co‐labeling experiments with fluorescently labeled beads and DAPI demonstrated the MFV and the vacuole containing the polyP body (and presumably associated Ca^2+^) are physically and functionally discrete compartments of the cell.

The biogenesis and eventual loss of the MFV will require further study. Acidocalcisomes are produced *de novo* from the Golgi apparatus in taxonomically distant protists (Goodenough *et al*., 2019), while the lytic vacuoles of plants may be either inherited or produced *de novo* depending on the cell type (Cui *et al*., 2020). The MFV of *S. apsteinii* is constitutively expressed and inherited during mitosis, a set of features most similar to the inheritance of vacuoles in yeast (Weisman, 2006). Due to the loss of the LTG signal during late stationary phase, we hypothesize that maintenance of the membrane integrity and pH gradient of the MFV were compromised; leakage from the MFV could trigger dramatic metabolic changes associated with senescence and characteristic of lysosomal cell death (Aits & Jäättelä, 2013; Wang *et al*., 2018).

Given its shared function for phagotrophy and autophagy, the MFV appears to be required for both nutrient assimilation and recycling and the regulation of growth. It is interesting to consider if the MFV represents an innovation that stems from the tertiary or quaternary endosymbiotic origins of the haptophytes (de Vargas *et al*., 2007; Stiller *et al*., 2014). The current view of marine mixotrophs is that they maintain a discrete phagocytic apparatus and food vacuole(s) (that can be stained by acidophilic dyes such as Lysotracker) as well as the photosynthetic machinery (Millette *et al*., 2024). Therefore, the costs of maintaining phototrophic and phagotrophic modes of carbon acquisition in constitutive mixotrophs is considered a critical tradeoff that limits growth rates (Millette *et al*., 2023). The selective pressure to retain phagotrophy may, however, have given rise to a single MFV that lowers the overall energetic cost to the constitutive mixoplankton by serving to degrade and recycle both endogenous and exogenous particulate organic carbon in a metabolically efficient manner.

To our knowledge these results are the first to reveal a functional convergence of two degrative cellular pathways (phagotrophy and chlorophagy) to a terminal digestive organelle in a photosynthetic organism. The presence of a constitutive acidic MFV highlights the need for caution when using acidophilic probes such as Lysotracker in high‐throughput assays to infer phagotrophy or grazing in natural populations of mixoplankton (Wilken *et al*., 2019). In the case of *S. apsteinii*, the presence of an acidic vacuole could be the result of autophagy in the absence of active phagotrophy; therefore, labeled prey particles are still needed to validate phagotrophy on a case‐by‐case basis.

The discovery of the MFV opens many new questions regarding how phagotrophy and autophagy are regulated in mixoplankton, such as the biochemical nature of the MFV, the mechanism of intracellular transport of cargo to the MFV, and its role in growth and senescence. The constitutive phagocytic and chlorophagic activity associated with the MFV of the coccolithophore implies a significant role for heterotrophic nutrition throughout growth as well as during periods of nutrient scarcity, and/or light limitation, or oxidative stress. Studies that investigate phagocytosis and chlorophagy of *S. apsteinii* in response to growth irradiance, prolonged darkness, and nutrient limitation are now needed to more clearly understand the physiological and ecological factors that regulate these pathways. The driving factors of mixotrophy are especially important to identify given the dominant contribution larger nonmotile diploid coccolithophore species make to particulate organic and inorganic carbon production and export in many biogeographic regions (Daniels *et al*., 2014, 2016; Rigual Hernández *et al*., 2020).

The nutritional role of phagocytosis in *S. apsteinii* can be considered as follows; With a carbon assimilation of 130 pg POC cell^−1^ d^−1^ for *S. apsteinii* (Gafar *et al*., 2019) then 1% of daily cellular C‐quota could be met by ingestion of *c*. 40 bacteria or *c*. 3 nanoflagellates assuming 30 fg C cell^−1^ for coastal heterotrophic bacteria (Fukuda *et al*., 1998), up to 200 fg cell^−1^ for a cyanobacteria (e.g. Procholorococcus), or 500 fg cell^−1^ for a picophytoplankton (Bertilsson *et al*., 2003; Barton *et al*., 2023). Measurements of bacterial grazing in motile freshwater cryptophyte algae with a similar biovolume to *S. apsteinii* determined that 2.1% and 8.5% of C and N quota d^−1^, respectively, were acquired through grazing rates of 8 bacteria cell^−1^ h^−1^ (Koppelle *et al*., 2024). Feeding experiments in natural populations of marine haptophytes in oligotrophic waters demonstrated the 5–20 μm size class ingested an average of 1.5 bacteria prey cell^−1^ h^−1^ yielding and estimated acquisition of 3.7% of daily carbon quota (Unrein *et al*., 2014). We can infer a potential ingestion rate of at least 1 prey particle cell^−1^ h^−1^ for *S. apsteinii*, based the accumulation of > 20 fluorescent beads in the MFV which, depending on the prey type, could provide for similar levels of nutrient acquisition in these calcified and nonmotile cells. We conclude phagotrophy of prey particles in diploid calcified and nonmotile *S. apsteinii* is nutritionally and ecologically relevant, potentially enabling them to acquire similar levels of C and essential elements (e.g. N, P, and trace metals) to flagellated noncalcified haptophytes.

How prevalent phagotrophy is among diploid calcifying coccolithophores remains to be determined. We found no evidence of active FluoSphere bead update or pH Rhodo Bioparticles in either *G. huxleyi* or *C. pelagicus* over 12 h, which is consistent with the very low incidence of fluorescently labeled bacteria uptake for calcified *G. huxleyi* and lack of uptake observed in *C. pelagicus* (Zubkov & Tarran, 2008; Avrahami & Frada, 2020; Ye *et al*., 2024), both diploid coccolithophores with interlocking coccoliths. In the case of *G. huxleyi*, its relatively small SA/V ratio may favor osmotrophy, as demonstrated in both laboratory and field measurements (Godrijan *et al*., 2020; Balch *et al*., 2023), over phagotrophy for supplementing C‐acquisition. It is increasingly clear that nutritional flexibility underpins the ecological success of haptophytes and is determined by a complex interplay of traits such as cell size, motility (i.e. potential prey encounter rate), calcification state, and coccolith type among diploid coccolithophores and between their alternating life phases, combined with nutrient availability that will govern the degree to which any given species relies on osmotrophy, phagotrophy, and autophototrophy.

In conclusion, we present definitive evidence that the nonmotile diploid coccolithophore *S. apsteinii* is a constitutive mixotroph, with observations of prey capture (most likely by filopodia) and processing within a single prominent acidic vacuole to which chloroplast fragments were also targeted for degradation. This MFV is inherited during mitosis and expressed throughout nutrient‐replete growth until shortly before senescence. The MFV is distinct from the primary storage compartment for calcium‐polyP. These observations establish the MFV as a novel organelle in the coccolithophores that is shared by the phagocytosis and autophagy pathways and is essential to nutrient acquisition and recycling and ultimately to growth and survival. Our work also highlights the need for continued efforts to characterize mixotrophy traits for a wide range of coccolithophores to better understand the C‐sink‐source state of coccolithophore communities in the oceans and in response to ocean warming.

## Competing interests

None declared.

## Author contributions

ART provided the conceptual framework and ART, OF and JAK planned and designed experiments. MBC, OF, JAK, ES and ART performed the experiments and data analysis, JAK conducted bioinformatics. JAK and ART wrote and edited the manuscript.

## Disclaimer

The New Phytologist Foundation remains neutral with regard to jurisdictional claims in maps and in any institutional affiliations.

## Supporting information


**Fig. S1** Validation of Lysotracker staining in *Scyphosphaera apsteinii*.
**Fig. S2** Representative examples of uptake and colocalization of 1 μm FluoSphere beads with pHrodo *Escherichia coli* in *Scyphosphaera apsteinii*.
**Fig. S3** Second experiment demonstrating presence of a Lysotracker Green (LGT)‐stained vacuole during *Scyphosphaera apsteinii* culture growth.
**Fig. S4** The digestive vacuole of *Scyphosphaera apsteinii* plays a central role in chlorophagy.
**Fig. S5** Additional TEM micrographs of *Scyphosphaera apsteinii* cells with ingested 1 μm beads and beads outside of cells.
**Fig. S6** TEM evidence of the constitutive degrative vacuole in control (‘unfed’) *Scyphosphaera apsteinii* cells.

**Tables S1–S4** Transcript and gene IDs for *Scyphosphaera apsteinii* and *Gephyrocapsa huxleyi* (as *Emiliania huxleyi*) associated with phagotrophy and autophagy pathways.
**Table S5** FluoSphere beads were not ingested by two other coccolithophore species, *Gephyrocapsa huxleyi* and *Coccolithus braarudii*.
**Table S6** Colocalization of Lysotracker Green (LTG) and ingested FluoSpheres in *Scyphosphaera apsteinii*.
**Table S7** Potential for chlorophagy in *Scyphosphaera apsteinii*.
**Tables S8–S11** KEGG Orthology (KO) numbers of the metabolic pathways associated with phagotrophy and autophagy and the number of *Scyphosphaera apsteinii* and *G. huxleyi* (as *Emiliania huxleyi*) transcripts and gene IDs identified for each.

**Video S1** Decalcified *Scyphosphaera apsteinii* interact with bacteria and particles through lamellopodia.

**Video S2** Normally calcified *Scyphosphaera apsteinii* extend filopodia from between muroliths.

**Video S3** Pigmented structures within the centralized (multifunctional) vacuole indicate chlorophagy in *Scyphosphaera apsteinii*.

**Video S4** Chlorophyll autofluorescence within the centralized (multifunctional) vacuole of *Scyphosphaera apsteinii* colocalizes with Lysotracker red staining.

**Video S5** Chlorophyll autofluorescence within the centralized (multifunctional) vacuole of *Scyphosphaera apsteinii* is discrete from the large lobed chloroplasts of *S. apsteinii*.
Please note: Wiley is not responsible for the content or functionality of any Supporting Information supplied by the authors. Any queries (other than missing material) should be directed to the *New Phytologist* Central Office.

## Data Availability

The data that support the findings of this study are openly available in Figshare at doi: 10.6084/m9.figshare.28046255.
