# Supplementary material for: A multifunctional organelle coordinates phagocytosis and chlorophagy in a marine eukaryote phytoplankton Scyphosphaera apsteinii
Source: New Phytol. 2025 Mar 4;246(3):1096–112. doi: 10.1111/nph.20388 (PMC11982794; doi:10.1111/nph.20388)
Supplement: Supplementary file 1 — Fig. S1 Validation of Lysotracker staining in Scyphosphaera apsteinii. Fig. S2 Representative examples of uptake and colocalization of 1 μm FluoSphere beads with pHrodo Escherichia coli in Scyphosphaera apsteinii. Fig. S3 Second experiment demonstrating presence of a Lysotracker Green (LGT)‐stained vacuole during Scyphosphaera apsteinii culture growth. Fig. S4 The digestive vacuole of Scyphosphaera apsteinii plays a central role in chlorophagy. Fig. S5 Additional TEM micrographs of Scyphosphaera apsteinii cells with ingested 1 μm beads and beads outside of cells. Fig. S6 TEM evidence of the constitutive degrative vacuole in control (‘unfed’) Scyphosphaera apsteinii cells. [file NPH-246-1096-s002.pdf]

***New Phytologist* Supporting Information**

Article title: **A multifunctional organelle coordinates phagocytosis and chlorophagy in a marine eukaryote phytoplankton *Scyphosphaera apsteinii.***
Authors: Julie A. Koester, Oren Fox, Elizabeth Smith, Madison B. Cox & Alison R. Taylor
Article acceptance date: 06 October 2024

The following Supporting Information is available for this article:

**Fig. S1** Validation of Lysotracker Staining in *S. apsteinii*.

**Fig. S2** Representative examples of uptake and colocalization of 1 μm FluoSphere beads with pHrodo *E. coli* in *S. apsteinii*.

**Fig. S3** Second experiment demonstrating presence of a Lysotracker Green (LGT)-stained vacuole during *Scyphosphaera apsteinii* culture growth.

**Fig. S4** The digestive vacuole of *S. apsteinii* plays a central role in chlorophagy.

**Fig. S5** Additional TEM micrographs of *S. apsteinii* cells with ingested 1 μm beads and beads outside of cells.

**Fig. S6** TEM evidence of the constitutive degrative vacuole in control ('unfed') *S. apsteinii* cells.

**Table S1-S4** Transcript and gene IDs for *Scyphosphaera apsteini* and *Gephyrocapsa huxleyi* (as *Emiliania huxleyii*) associated with phagotrophy and autophagy pathways (as Excel Workbook).

**Table S5** Fluosphere beads were not ingested by two other coccolithophore species.

**Table S6** Colocalization of LTG and ingested Fluospheres

**Table S7** Potential for chlorophagy in *S. apsteinii*.

**Table S8-S11** KEGG Orthology (KO) numbers of the metabolic pathways associated with phagotrophy and autophagy and the number of *S. apsteinii* and *G. huxleyi* (as *Emiliania huxleyi*) transcripts and gene IDs identified for each.

**Video/Movie S1** Decalcified *S.apsteinii* interact with bacteria and particles through lamellopodia. Monochrome phase contrast time lapse movie showing movement of decalcified cell on a coverslip as it interacts and appears to ingest bacteria. Length of movie represents approximately 2h.

**Video/Movie S2** Normally calcified *S.apsteinii* extend filopodia from between muroliths. Color DIC time lapse movie showing filopodia movement at the cell surface that are extending well beyond the calcified coccosphere. Movie represents approximately 30 min.

**Video/Movie S3** Pigmented structures within the centralized (multifunctional) vacuole indicate chlorophagy. Color widefield DIC Z-stack sequence of images showing fragment of chloroplast within vacuole structure (arrow). See Fig. **8 a,b**.

**Video/Movie S4** Chlorophyll autofluorescence within the centralized (multifunctional) vacuole of *S.apsteinii* colocalizes with lysotracker red staining. Movie shows a confocal Z-stack sequence of 4 cells (see Fig. **S4 c-d**) with overlay of DIC, chlorophyll (green) fluorescence and Lysotracker fluorescence (red) channels.

**Video/Movie S5** Chlorophyll autofluorescence within the centralized (multifunctional) vacuole of *S.apsteinii* is discrete from the large lobed chloroplasts of *S.apsteinii.* This movie shows the volume rendered confocal Z-stack of Chlorophyll fluorescence (green) and Lysotracker fluorescence (red) of the 4 cells in Fig. **S4 c-d**.

**Fig. S1 Validation of Lysotracker Staining in *S. apsteinii***. **(a,b)** Confocal micrographs of control (unstained) cells in the absence of Lysotracker Green (LTG) showing only chlorophyll (Chl) autofluorescence (red). **(c,d)** Confocal micrographs of cells treated with Lysotracker Green with the same acquisition settings as (a,b) showing brightly fluorescent lysotracker positive vacuoles (green). Scale bars = 25 μm. **(e)** Flow cytometry dot plot and **(f)** population histogram of 10,000 Lysotracker Green labelled *S. apsteinii* with distribution statistics from each respective gate. **(g)** Normalized histogram plots showing the shift in BB515-A channel signal (Lysotracker) with increasing Lysotracker concentration. Note that in the absence of LTG, the cells exhibit autofluorescence (due to detection of pigments and degradation products of chlorophyll in the BB515-A channel) requiring gating for LTG positive cells. **(h)** Comparison of LTG positive cell populations assessed through confocal microscopy and flow cytometry (error bars = SD). The 50 nM Lysotracker concentration was chosen for all subsequent experiments.

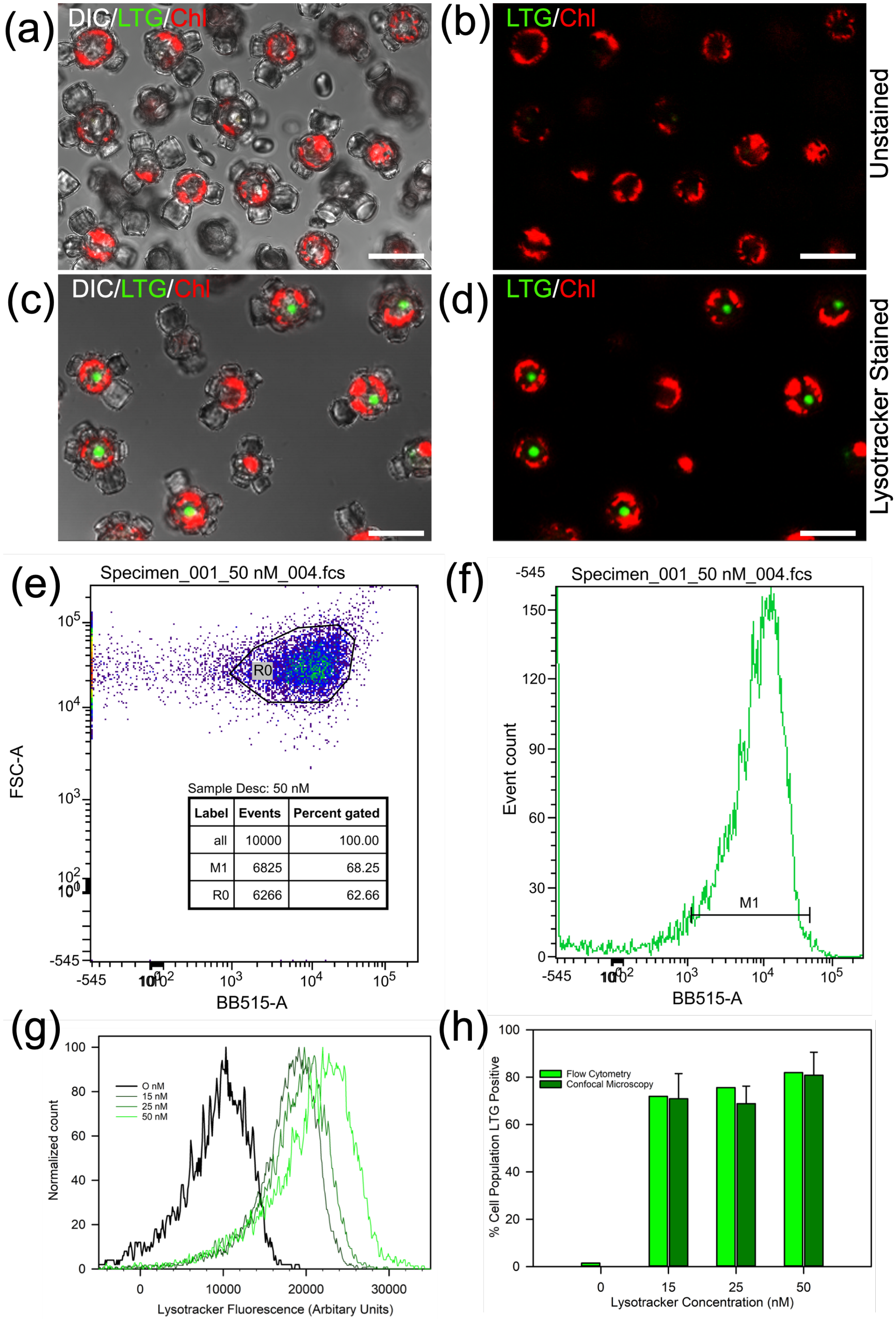

(a)
DIC/LTG/Chl
(b)
LTG/Chl
Unstained
(c)
DIC/LTG/Chl
(d)
LTG/Chl
Lysotracker Stained
(e)
Specimen_001_50 nM_004.fcs
R0
Sample Desc: 50 nM
Label | Events | Percent gated
all | 10000 | 100.00
M1 | 6825 | 68.25
R0 | 6266 | 62.66
FSC-A
BB515-A
(f)
Specimen_001_50 nM_004.fcs
M1
Event count
BB515-A
(g)
O nM
15 nM
25 nM
50 nM
Normalized count
Lysotracker Fluorescence (Arbitary Units)
(h)
Flow Cytometry
Confocal Microscopy
% Cell Population LTG Positive
Lysotracker Concentration (nM)

**Fig. S2 Representative examples of uptake and colocalization of 1 μm FluoSphere beads with pHrodo *E. coli* in *S. apsteinii*.** **(a)** clockwise from top left a Z-series of confocal sections from lower surface of a cell to upper surface showing a cluster of 1 μm NR beads (red) located inside the cell. **(b-d)** Lower, mid, and upper DIC overlay confocal sections through a cell in which Y FluoSphere beads (green) colocalize with the MFV which fluoresces red due to ingestion and processing of the *E. coli* cells at low pH, yellow = colocalization signal. **(e-h)** Single confocal section through a cell that has ingested yellow green FluoSphere **(f)** and pHrodo *E. coli* **(g)** that are processed in the same acidic vacuole (yellow = colocalization signal). **(h)** DIC overlay of all channels. Scale bars = 10 μm.

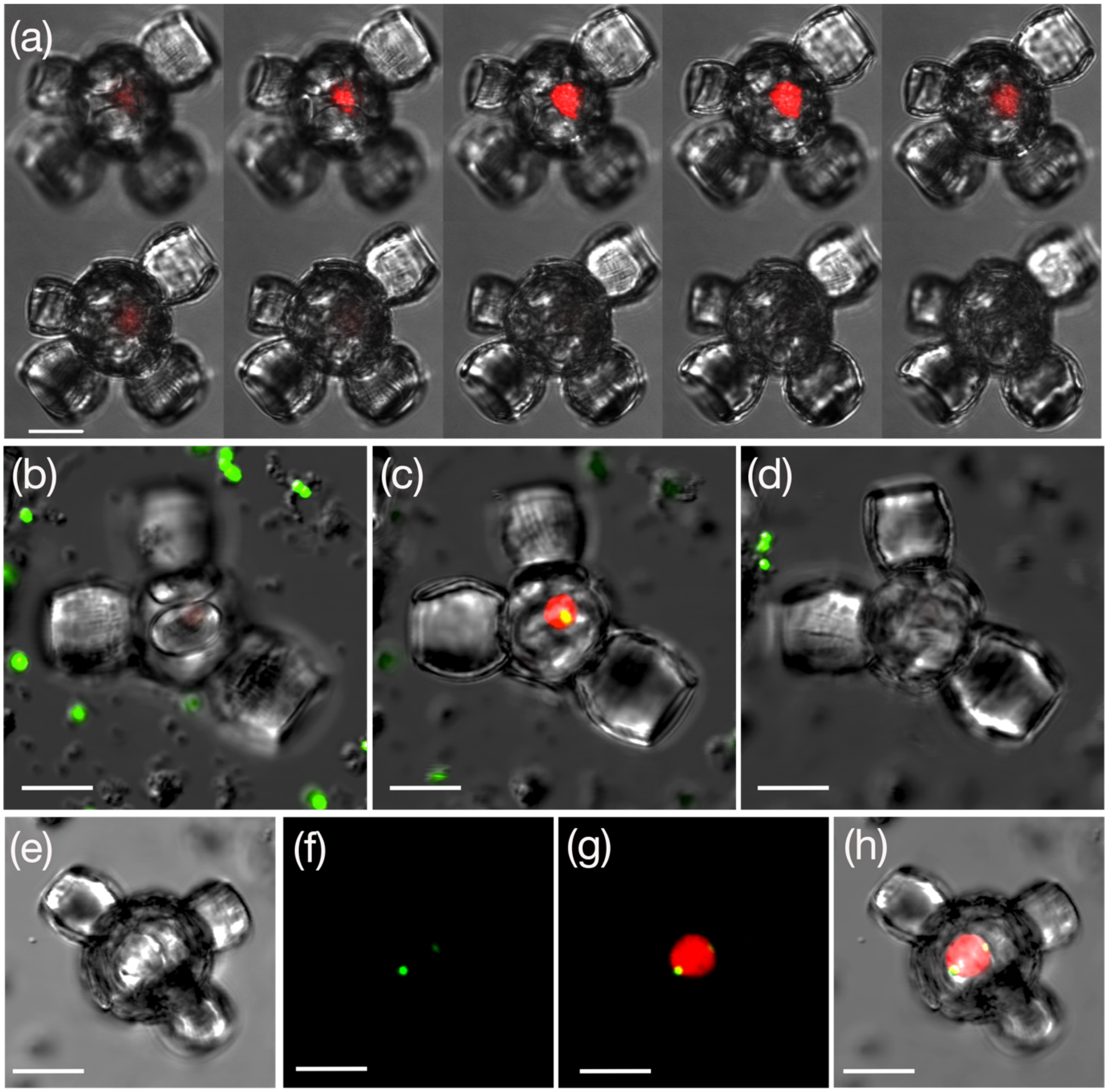
(a)
(b)
(c)
(d)
(e)
(f)
(g)
(h)

**Fig. S3 Second experiment demonstrating presence of a Lysotracker Green (LGT)-stained vacuole during *S. apsteinii* culture growth**. The proportion of LTG positive cells was determined by both confocal microscopy and flow cytometry on replicate batch cultures (n = 3) yielding comparable results. Photosynthetic physiology (Fv/Fm) was determined from 10 d onwards at which point the cell density was high enough to yield a sufficient chlorophyll fluorescence signal in a 1 mL sample of cells. A decline in LTG positive cells is evident at 15 d while cell growth and Fv/Fm remain stable. This supports the hypothesis that the MFV biochemistry changes prior to population senescence and cell death.

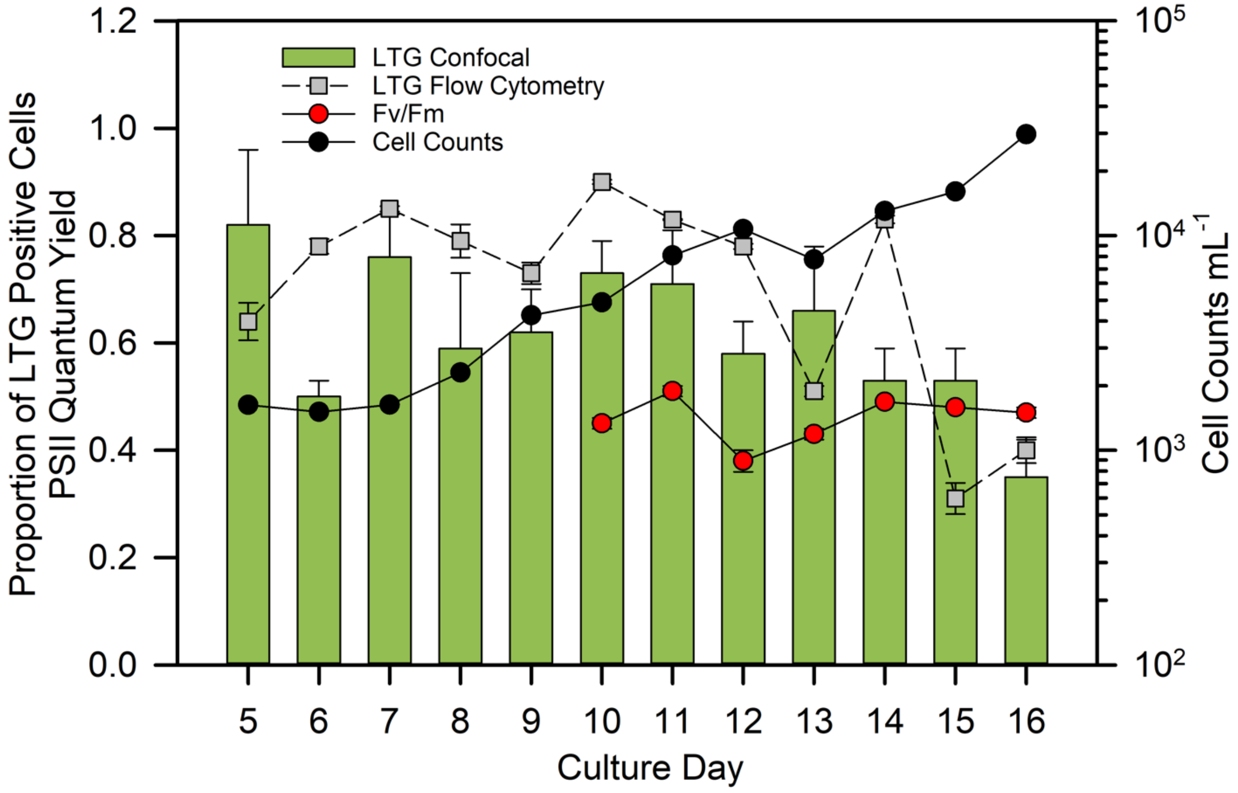

LTG Confocal
LTG Flow Cytometry
Fv/Fm
Cell Counts
Proportion of LTG Positive Cells
PSII Quantum Yield
Cell Counts mL$^{-1}$
Culture Day
1.2
1.0
0.8
0.6
0.4
0.2
0.0
$10^5$
$10^4$
$10^3$
$10^2$
5
6
7
8
9
10
11
12
13
14
15
16

**Fig. S4 The digestive vacuole of *S. apsteinii* plays a central role in chlorophagy. (a,b)** Brightfield and DIC images of a decalcified *S. apsteinii* cell showing prominent vacuole (black arrows) with dense pigmented chloroplast fragments within. **(c-f)** Confocal maximum projection of 4 cells stained with 50 nM Lysotracker red to label the acidic multifunctional vacuole. Three of the 4 cells have a prominent vacuole (dotted circles) that also exhibits punctate chlorophyll signal closely colocalized within. Please refer to Supplementary movies **S4** and **S5** for corresponding Z-stack and 3D rendering of these data. Overall, the two fluorescent channels demonstrate the physical colocalization of chlorophyll autofluorescent fragments and the LTR stained vacuoles. Scale bars = 10 μm.

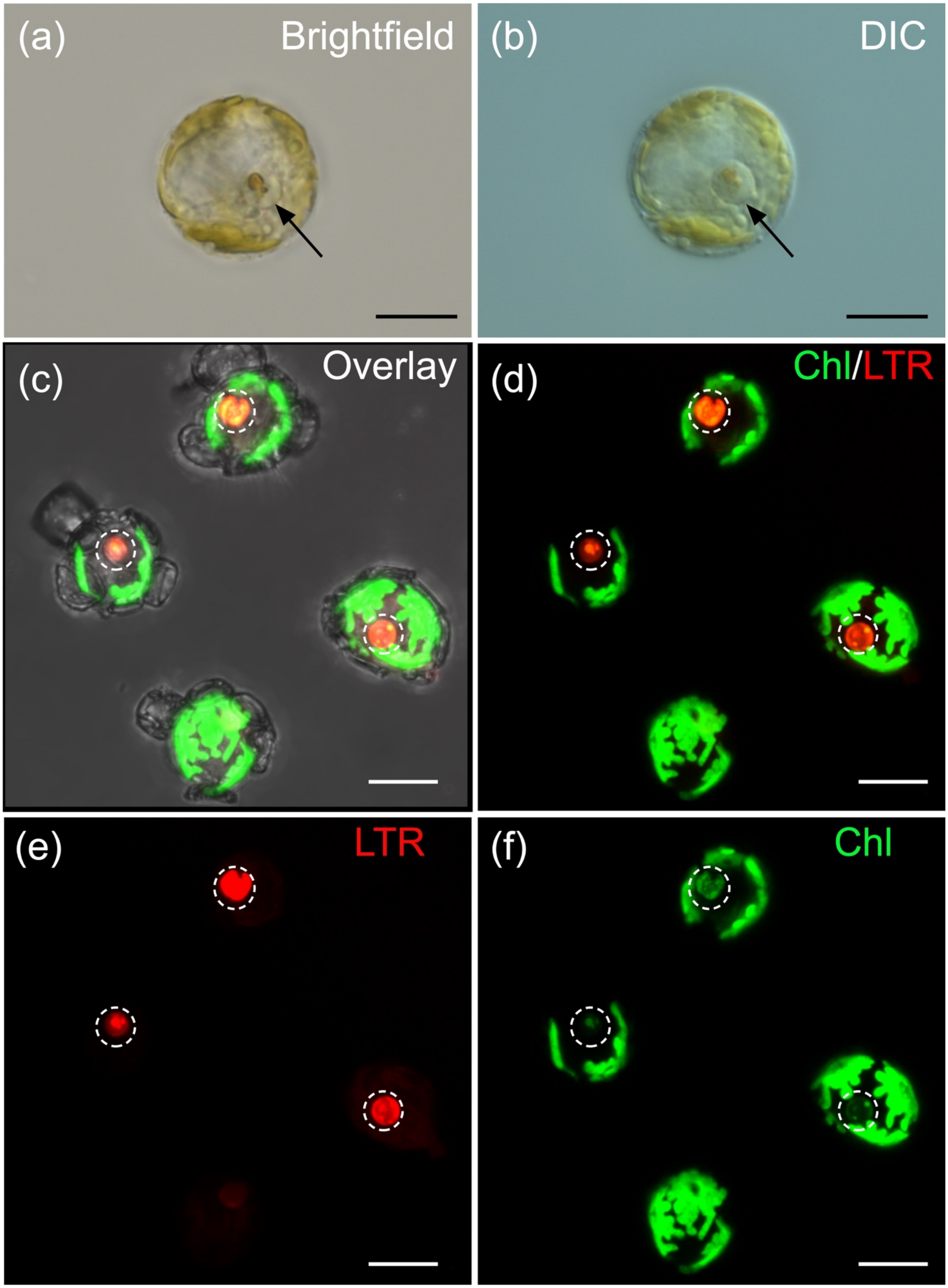

(a)
Brightfield
(b)
DIC
(c)
Overlay
(d)
Chl/LTR
(e)
LTR
(f)
Chl

**Fig. S5 Additional TEM micrographs of *S. apsteinii* cells with ingested 1 μm beads and beads outside of cells**. **(a-d)** Whole cell and detail showing a tightly packed multi-functional vacuole (MFV) closely associated with the nucleus (N) containing multiple 1 μm beads in a matrix of darkly stained amorphous material (p), likely a suite of enzymes and other proteins related to degradation. The beads (*) have undergone significant degradation. Mu and Lo are holes in the resin left on dissolution of the external murolith and lopadolith coccoliths, respectively. Chl = chloroplast, M = mitochondria. **(e,f)** Examples of 1 μm beads in the resin outside of cells that have not been ingested or degraded. Scales: 10 μm for a,c and 1 μm for b,d,e,f.

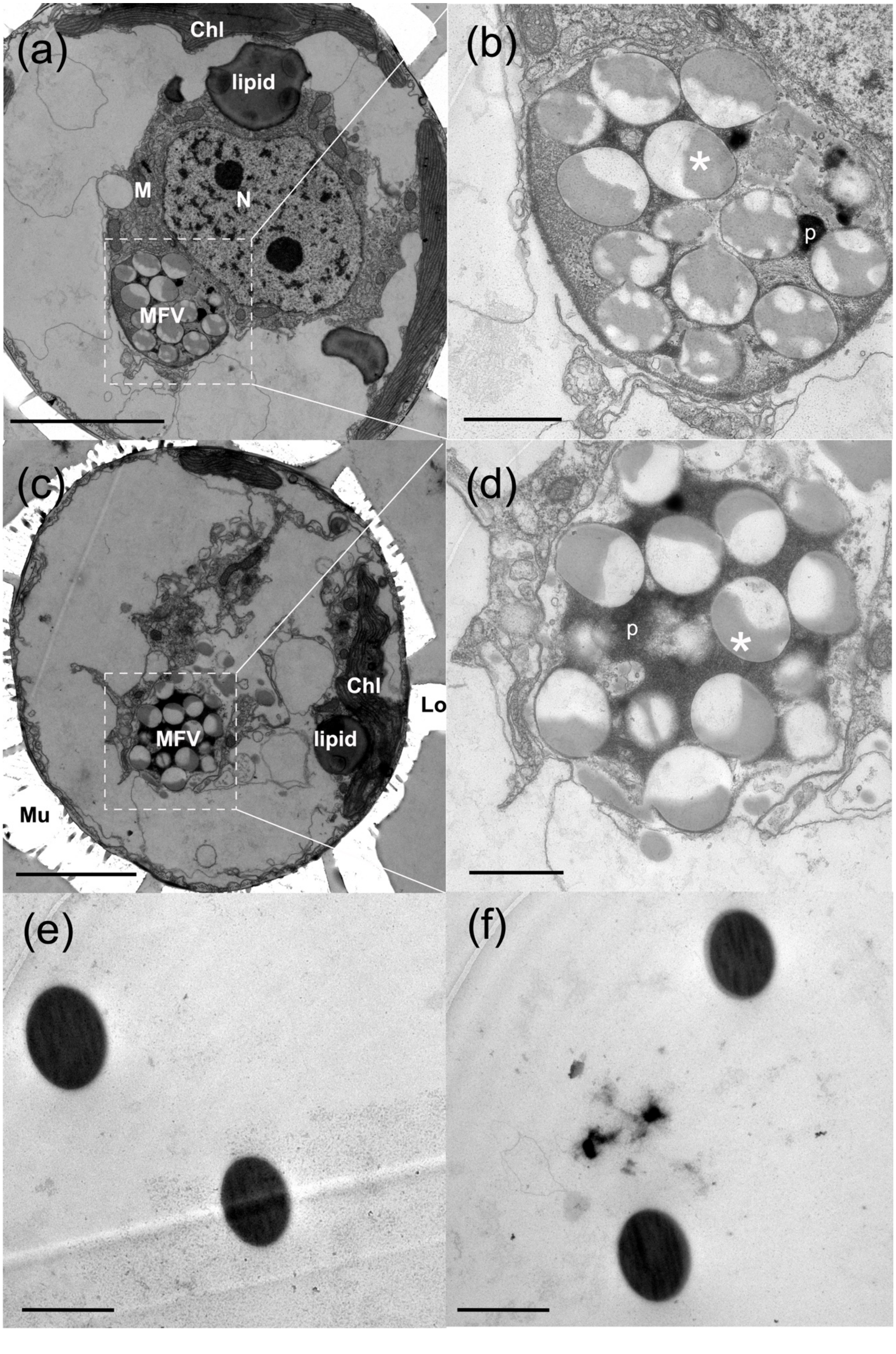
(a)
Chl
lipid
M
N
MFV
(b)
*
p
(c)
Chl
Lo
lipid
MFV
Mu
(d)
p
*
(e)
(f)

**Fig. S6 TEM evidence of the constitutive degrative vacuole in control ('unfed') *S. apsteinii* cells**. **(a,b)** Control (no added prey particles) cells with constitutive digestive vacuole (highlighted in pale blue), adjacent to the nucleus (N) at the periphery of the cytoplasmic core, between the nucleus and the vacuolar space (V). A variety of diffuse and punctate organic material is evident within the degrative vacuole. Lopadolith (Lo) and Murolith (Mu) coccoliths are seen as gaps in the resin on the outside of the cells where calcite has dissolved. In **(b)** a murolith at the point of secretion is seen (*) as a new baseplate scale commences mineralization (black arrows). An additional vacuole with darkly stained material is also seen (highlighted in pink) that may represent the polyP storage vacuole. **(c-h)** 3 additional whole cell micrographs of the presumptive digestive vacuole highlighted in pale blue; dotted arrows indicate the detailed features in the accompanying panel to the right. Mitochondria (M), chloroplasts (Ch) and Golgi body (G) are indicated where appropriate.

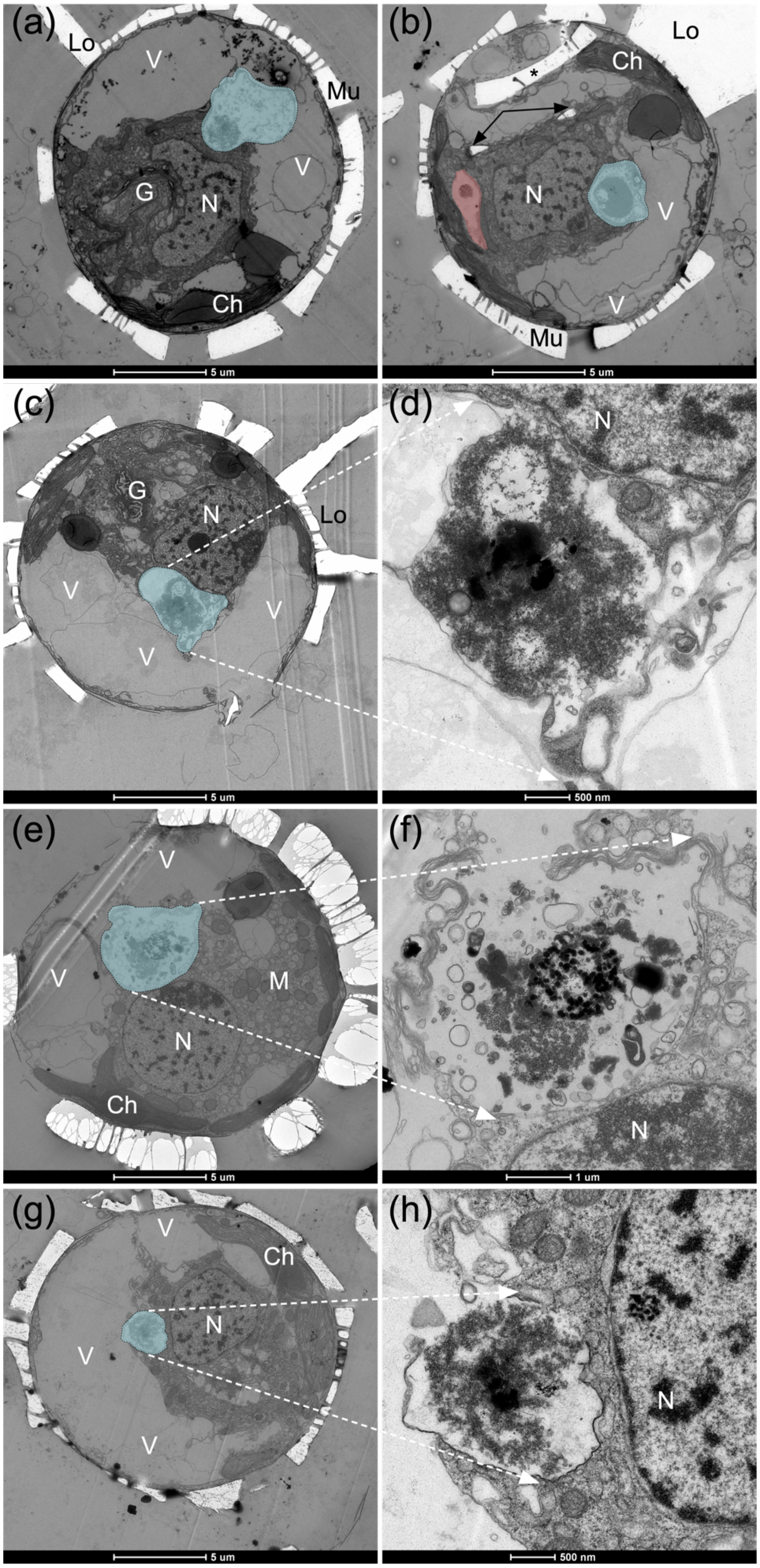

(a)
Lo
V
Mu
V
G
N
Ch
5 um
(b)
Lo
Ch
*
N
V
V
Mu
5 um
(c)
G
N
Lo
V
V
V
5 um
(d)
N
500 nm
(e)
V
V
M
N
Ch
5 um
(f)
N
1 um
(g)
V
Ch
N
V
V
5 um
(h)
N
500 nm

**Table S1-S4** Transcript and gene IDs for *Scyphosphaera apsteinii* and *Gephyrocapsa huxleyii* (as *Emiliania huxleyii*) associated with phagotrophy and autophagy pathways (as Excel Workbook). These transcripts were used to develop the conceptual model in Fig. **10**.

**Table S5 Fluosphere beads were not ingested by two other coccolithophore species.** Replicate (n = 3) cultures of *Gephyrocapsa huxleyii* and *Coccolithus braarudii* were incubated with 0.5 μm, 1.0 μm, and in case of *C. braarudii*, 2.0 μm beads that were both untreated and Methylcellulose-treated. Untreated beads were carboxylate-modified and negatively charged, and MC treated bead carried a neutral charge. Regardless of size or surface treatment, no beads were detected inside cells. Light decalcification was used to enhance possibility of detection. We conclude that ingestion of particles in these 2N cells does not occur or is very uncommon. A summary of the total cells scored in the 3 replicated experiments is given below.

| | Treatment | Fluosphere size and color | Total # cells scored | Cells with ingested fluosphere | # Experiments |
|---|---|---|---|---|---|
| ***C. braarudii*** | | | | | |
| | Methyl Cellulose | Y 0.5 μm | 282 | 0 | n=3 |
| | | Y 1.0 μm | 159 | 0 | n=3 |
| | | Y 2.0 μm | 62 | 0 | n=2 |
| | Untreated | Y 0.5 μm | 267 | 0 | n=3 |
| | | Y 1.0 μm | 307 | 0 | n=3 |
| | | Y 2.0 μm | 141 | 0 | n=2 |
| ***G. huxleyi*** | | | | | |
| | Methyl Cellulose | Y 0.5 μm | 773 | 0 | n=3 |
| | | Y 1.0 μm | 778 | 0 | n=3 |
| | Untreated | Y 0.5 μm | 1020 | 0 | n=3 |
| | | Y 1.0 μm | 555 | 0 | n=3 |

**Table S6 Colocalization of LTG and ingested Fluospheres**. In this experiment *S. apsteinii* cells were incubated with 1 μm untreated (UT) Nile Red Fluospheres and subsequently stained and scored for the presence of an LTG-stained acidic vacuole, bead uptake, and colocalization of Fluospheres and LTG.

| | Total Cells | positive | % cells |
|---|---|---|---|
| Lysotracker | 68 | 49 | 72.1 |
| Lysotracker with beads | 113 | 67 | 59.3 |
| 3 h 1 μm UT bead uptake | 113 | 17 | 15.0 |
| Colocalized (LGT & Bead) | 17 | 11 | 64.7 |

**Table S7 Potential for chlorophagy in *S. apsteinii*.** The table presents the frequency of autofluorescent chlorophyll fragments observed within the lysotracker positive vacuole. Three independent experiments were conducted on late exponential to early stationary cultures by staining aliquots of cells with 50 nM Lysotracker Red and confocal imaging Z-stacks to assess frequency of the lysotracker stained acidic vacuole and colocalization of chlorophyll autofluorescence that indicates uptake of chloroplast fragments.

| Experiment | Cells scored | % Cells with LTR MFV | % MFV with Chloroplast | % Chlorophagic Cells |
|---|---|---|---|---|
| 1 | 118 | 65.3 | 54.5 | 35.6 |
| 2 | 209 | 67.9 | 63.4 | 43.1 |
| 3 | 116 | 46.6 | 50.0 | 23.3 |
| **Average** | **148** | **59.9%** | **56.0%** | **34.0%** |
| StDev | | 11.7 | 6.8 | 10.0 |
| N experiments | | 3 | 3 | 3 |

**Table S8-S11** KEGG Orthology (KO) numbers of the metabolic pathways associated with phagotrophy and autophagy and the number of *Scyphosphaera apsteinii* and *Gephyrocapsa huxleyi* (as *Emiliania huxleyi*) transcripts and gene IDs identified for each. See Excel workbook.
